# Dual-color terahertz spatial light modulator for single-pixel imaging

**DOI:** 10.1038/s41377-022-00879-5

**Published:** 2022-06-23

**Authors:** Weili Li, Xuemei Hu, Jingbo Wu, Kebin Fan, Benwen Chen, Caihong Zhang, Wei Hu, Xun Cao, Biaobing Jin, Yanqing Lu, Jian Chen, Peiheng Wu

**Affiliations:** 1 Research Institute of Superconductor Electronics (RISE), School of Electronic Science and Engineering, Nanjing University, Nanjing, 210023 China; 2 School of Electronic Science and Engineering, Nanjing University, Nanjing, 210023 China; 3 Purple Mountain Laboratories, Nanjing, 211111 China; 4 National Laboratory of Solid State Microstructures, Collaborative Innovation Center of Advanced Microstructures and College of Engineering and Applied Sciences, Nanjing University, 163 Xianlin Avenue, Nanjing, 210023 China

**Keywords:** Liquid crystals, Terahertz optics

## Abstract

Spatial light modulators (SLM), capable of dynamically and spatially manipulating electromagnetic waves, have reshaped modern life in projection display and remote sensing. The progress of SLM will expedite next-generation communication and biomedical imaging in the terahertz (THz) range. However, most current THz SLMs are adapted from optical alternatives that still need improvement in terms of uniformity, speed, and bandwidth. Here, we designed, fabricated, and characterized an 8 × 8 THz SLM based on tunable liquid crystal metamaterial absorbers for THz single-pixel compressive imaging. We demonstrated dual-color compressive sensing (CS) imaging for dispersive objects utilizing the large frequency shift controlled by an external electric field. We developed auto-calibrated compressive sensing (ACS) algorithm to mitigate the impact of the spatially nonuniform THz incident beam and pixel modulation, which significantly improves the fidelity of reconstructed images. Furthermore, the complementary modulation at two absorption frequencies enables Hadamard masks with negative element values to be realized by frequency-switching, thereby halving the imaging time. The demonstrated imaging system paves a new route for THz single-pixel multispectral imaging with high reliability and low cost.

## Abstract (web summary)

Liquid crystal metasurface based spatial light modulator is developed for terahertz dual-color compressive imaging. Significant improvement of imaging quality and speed is demonstrated using proposed auto-calibrated algorithms and frequency-switching method.

## Graphical abstract

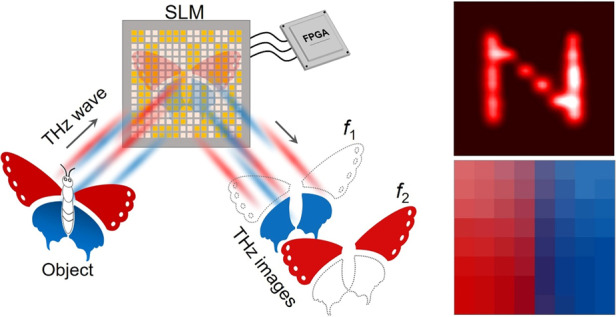

## Introduction

Spectral fingerprints of materials in the terahertz (THz) range hold a myriad of captivating light–matter interactions for physics and material science, such as lattice vibration, molecular rotation, and spin waves^1–4^. On the other hand, noninvasive identification of these signatures with spectral and spatial information enables numerous potential applications in biomedical diagnostic, pharmaceutical industry, and security inspection^5–7^. In recent years, there have been numerous efforts to develop hyperspectral or multispectral THz imaging techniques, such as coherent receiver array and THz time-domain spectroscopy (TDS)^8–10^. However, these techniques require complicated and high-cost equipment, limiting their widespread application.

Recently, single-pixel compressive imaging provides a route to achieve THz imaging at a lower cost^11^. The spatially modulated THz beam is collected by a bucket detector in the imaging setup, in which the spatial light modulators (SLMs) play an essential role. SLMs capable of dynamically modulating the phase^12^, amplitude^13^, and polarization of the light have already shown significant advantages in imaging, holography, and multiple-input multiple-output (MIMO) communications^14–18^. The commercially available SLMs operating in the optical regime, such as digital micromirror devices (DMD), can be directly adapted to the THz range for imaging by encoding a pumped beam incident on an optically active medium such as silicon and vanadium dioxide^19–23^. Pioneering demonstration of THz SLM based on metamaterial modulators has been used for THz single-pixel imaging^24,25^. Multispectral and hyperspectral single-pixel imaging require significant modulation over a wide spectral range^26,27^. However, the ohmic loss from the highly doped semiconductor thin-film damps the resonance significantly, leading to a slight frequency shift and a low amplitude modulation depth even at a high bias voltage. Up to date, THz SLM with a significant frequency shift and large modulation depths at multiple frequencies has not been demonstrated yet. Instead, due to the small loss tangent and large birefringence in the THz frequencies^28–30^, liquid crystals (LC) THz modulators have attracted tremendous interest for beam steering and imaging applications^31–33^. Furthermore, the LC-based THz devices are compatible with the industrial production line, which has been very mature for display and personal portable devices.

In conjunction with SLMs, computational imaging techniques, such as compressive sensing (CS), have been successfully applied for single-pixel imaging over the electromagnetic spectra from X-ray, visible light, and near-infrared to THz^34–38^. The main focus of the CS algorithm is to introduce more sophisticated image priors to further improve the reconstruction quality or reconstruction efficiency^39^. Under actual circumstances, however, the nonuniform distribution of illumination beam and the deviation of pixel modulation depths could seriously limit imaging quality for applications. However, the elimination of these nonuniformities has encountered technological challenges. On the one hand, it is laborious to enhance the uniformity of the SLM by improving the fabrication technique, especially for large-scale arrays. On the other hand, the nonuniformity of the THz source depends on the surrounding electromagnetic environment and is unknown until after measurement. When CS is introduced in specific applications, it offers the possibility of calibrating the non-idealness of the imaging system in the imaging reconstruction.

In this paper, utilizing the broadband frequency-switching of the LC metasurface absorber, we experimentally demonstrated an 8 × 8 SLM for dual-color single-pixel imaging. We formulated the non-idealness in our imaging system and proposed an auto-calibrated CS (ACS) method for higher-quality target object reconstruction. On this basis, we realized CS imaging of THz samples with dispersive characteristics. In addition, we used frequency-switching to achieve measurement with complementary coding patterns for nondispersive objects, which can reduce the imaging time by nearly 50%.

## Results

### Concept and design of dual-color THz SLM

**Fig. 1:**
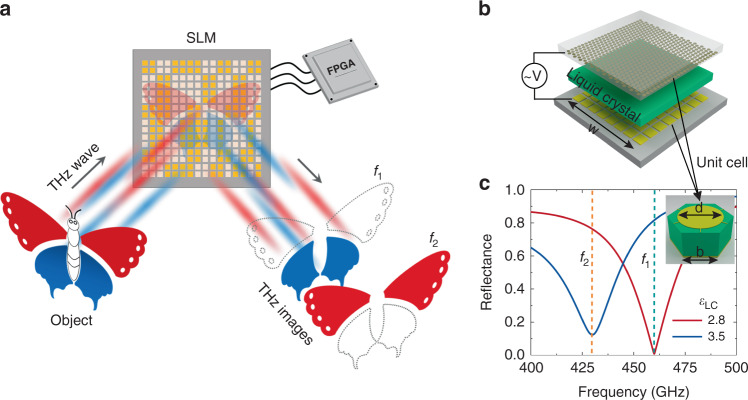
Working principle and design of the dual-color THz SLM. **a** Schematic diagram of the dual-color THz SLM. **b** Exploded view of the THz SLM. The resonant structures are on the back of the top quartz substrate, and the pixelated gold patches are on the front of the bottom quartz substrate. The thicknesses of the upper and lower quartz substrate are 300 and 500 μm, respectively, the thickness of the LC layer is 10 μm, and the side length of SLM (*w*) is 19.7 mm. **c** Simulated reflectance spectra of the SLM for different permittivities of LC (*ε*_LC_). The inset is the unit cell of the MMA with *d* = 240 μm and *b* = 173 μm

The dual-color THz SLM based on LC is conceptually illustrated in Fig. 1a. The upper and lower wings of a butterfly reflect THz waves at *f*_1_ and *f*_2_, respectively, so their spectral responses, i.e., colors, are different. To obtain the spectral information of the object, we used a continuous wave THz source^40^. The two THz beams with frequencies of *f*_1_ and *f*_2_ incident on the SLM subsequently can be spatially encoded by the SLM. The reflected beam from the SLM is focused and collected by a single-element detector. The reflection amplitudes of the SLM pixels at the two frequencies are different, so the received signals at the two frequencies can be used to reconstruct the object image. Since SLM works independently at *f*_1_ and *f*_2_, we can carry out single-pixel imaging for objects at *f*_1_ and *f*_2_, respectively, and obtain the spectral images after imaging reconstruction. To achieve this goal, we designed a THz SLM based on LC metasurface absorbers (MMA), consisting of 8 × 8 pixels with each pixel electrically controlled by a field-programmable gate array (FPGA).

Figure 1b shows the exploded diagram of the designed THz LC SLM. The LC layer is sandwiched between a metallic metasurface and a metallic ground layer. A 10-μm-thick dual-frequency LC (Jiangsu Hecheng Display Technology, DP002-016) was selected as the spacer, as it exhibits a large birefringence at 0.2–1.1 THz and does not need an alignment layer to define the orientation of LC molecules^41^. The metallic structures were patterned onto two quartz substrates, respectively. Metallic structures with a high filling factor are crucial for driving as many LC molecules as possible under the electric field. As shown in the inset of Fig. 1c, the unit cell of the metasurface in our design is a hexagonal lattice with a side length of *b* = 173 μm, and the resonator is a metallic disk with a diameter of *d* = 240 μm. The corresponding filling factor is 58%. Each disk is connected to the surrounding disks through the 2-μm-wide metallic wires. The pixelation of SLM was achieved by dividing the ground layer into 2.33 mm × 2.33 mm patches. The total area of the device is 19.7 mm × 19.7 mm. According to the measurement using THz time-domain spectroscopy as shown in Fig. S1^42^, the measured LC permittivity with and without electric field are 2.8 and 3.5, respectively. Meanwhile, the loss tangent is negligible. After taking the measured parameters into the simulation, the metasurface absorber exhibits a remarkable frequency redshift from 460 to 429.5 GHz, as shown in Fig. 1c.

### THz LC SLM for projection display

**Fig. 2:**
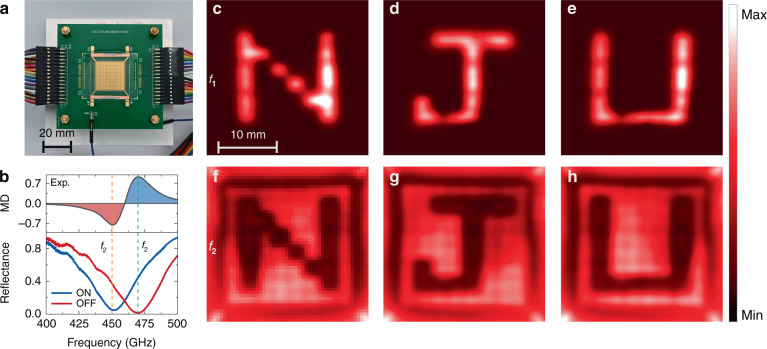
Fabricated dual-color THz SLM and characterization for projection display. **a** Photo of the fabricated THz SLM. **b** Measured reflectance spectra after smoothing and MD of the THz SLM. The blue curve represents the biased state with an amplitude of 10 V and a frequency of 1 kHz, and the red curve represents the state without bias. **c**–**e** are the projection display results at *f*_1_. **f**–**h** are the projection display results at *f*_2_

The fabricated THz LC SLM sample is shown in Fig. 2a (see methods for fabrication process). Figure 2b shows the measured reflection spectra with the applied bias of 0 (OFF) and 10 V (ON), respectively. The measured resonance frequency shifts from *f*_1_ = 470.2 to *f*_2_ = 450.7 GHz with a nearly 20 GHz frequency shift. The discrepancy between the experiments and the simulations is mainly caused by the deviation of the actual thickness of the LC layer. The corresponding modulation depth (MD) is defined as:^43^

1 $${\mathrm{MD}} = \frac{{R_{{\mathrm{ON}}} - R_{{\mathrm{OFF}}}}}{{R_{{\mathrm{ON}}} + R_{{\mathrm{OFF}}}}}$$

where *R*_ON_ and *R*_OFF_ are the reflection coefficients in the ON and OFF states, respectively. We measured the reflection spectra of the fabricated device by sweeping the frequency of the continuous wave THz source, which consists of a microwave signal generator (Keysight E8257D) and THz extension modules (VDI WR9.0M-SGX, WR4.3 × 2, and WR2.2 × 2). The reflected signal from the device is collected by a zero-bias Schottky diode detector (VDI WR2.2-ZBD). Figure 2b shows the measured reflection spectra after smoothing with the applied bias of 0 V (OFF) and 10 V (ON), respectively. The MD of the tunable metasurface absorber can reach higher than 70% at *f*_1_ and *f*_2_. Therefore, to obtain a better signal-to-noise ratio (SNR), we set the working frequency at these two frequencies for the following two-color imaging.

To verify the spatial modulation performance of the SLM, we controlled it as a programmable projection display. For displaying the letters “N”, “J”, and “U”, the pixels on the letter are biased with a 1 kHz bipolar square wave with an amplitude of 10 V, and other pixels are grounded. When the THz beam incident onto the LC SLM, the spatial patterns can be mapped out at the imaging plane with the raster scanning (see Fig. S5 for details of the experimental setup). The measurements at two frequencies were carried out separately by switching the frequency of the continuous wave THz source. Because of the distinct reflection for the pixels in ON and OFF states, as shown in Fig. 2b, the obtained images at *f*_1_ and *f*_2_ are complementary. As shown in Fig. 2c–e, the amplitude of letter parts is larger than that of the blank parts at *f*_1_. In contrast, complementary patterns are obtained, as shown in Fig. 2f–h. It can be seen that the projection images are not uniform, which is mainly due to the reflection variation of the SLM pixels caused by the uneven spacing between the top and bottom electrodes from the fabrication and packaging process. The experimental results indicate that the THz SLM can modulate the spatial THz field at two frequencies in a programmable way.

### Dual-color single-pixel imaging

Single-pixel imaging can be used to obtain images via interrogating the scene with a series of spatially controlled patterns funneled into a single-element detector. An object after pixelation can be described as a column vector *X*, including *N* elements. In the *i*th measurement, an interrogation mask with a row vector *Φ*_*ij*_ is multiplexed with the object, and a sensitive detector collects the signal with a value of *y*_*i*_. Then the whole procedure after total *M* measurements (*M* ≤ *N*) can be formalized as:^19^

2 $$y_i = \mathop {\sum}\nolimits_{j = 1}^N {\phi _{ij}x_j}$$

or the matrix description is *Y* = Φ×*X*, where *Y* ∈ ${\Bbb R}$^M×1^, ${{{\mathrm{{\Phi}}}}} \in {\Bbb R}^{M \times N}$, and $X \in {\Bbb R}^{N \times 1}$. When *M* < *N*, the inverse problem becomes ill-posed. The CS technique provides an efficient approach to reconstruct the image with a sparse representation.

Noises accompanying the measured data are inevitable due to the fluctuation of the source, vibration of the imaging system, and electronic readout noises. In an actual imaging system, the THz beams are not uniformly distributed on the object as shown in Fig. S8. In addition, the MD over the pixels is not even due to the fabrication tolerance. If a standard optimization algorithm is used for reconstruction without considering the nonuniformity, the extra error will be imposed onto the measurement masks. Consequently, the recovered image could deteriorate severely. To alleviate these noises and mitigate the artifacts from the nonuniformity, we introduced an ACS algorithm with the optimization target. In the ACS algorithms, we formulate the nonuniform factors, including the nonuniform pixel reflectance of the SLM and the nonuniform THz source intensity, in the imaging system. Through solving the proposed nonuniformity-involved imaging model, the nonuniform factors can be resolved by the proposed algorithm, achieving the auto-calibration of the imaging system and the image reconstruction simultaneously (see Methods for details).

**Fig. 3:**
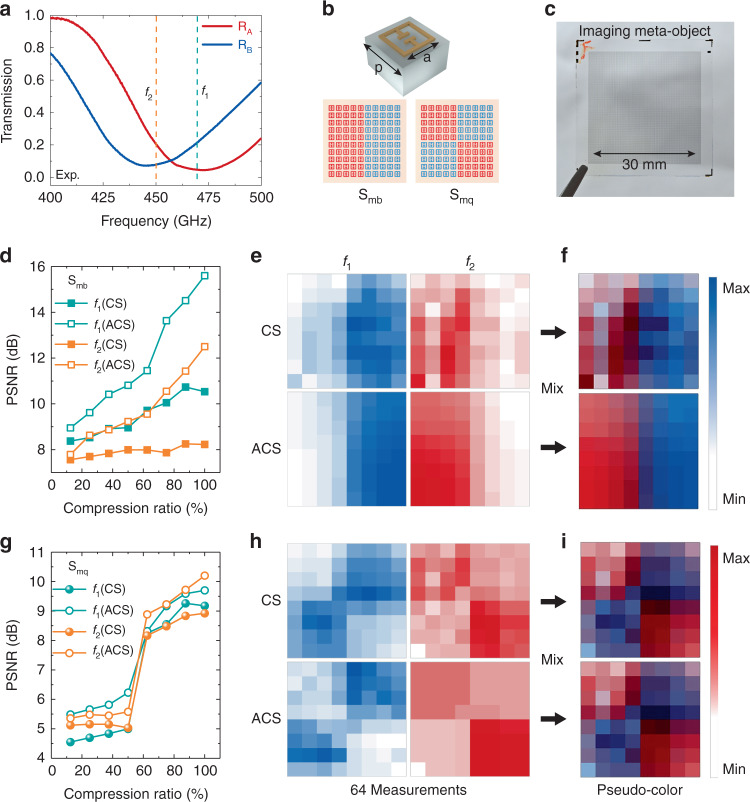
Dual-color imaging for THz dispersive meta-object with CS method. **a** Measured transmission spectra of R_A_ (red) and R_B_ (blue) after smoothing. **b** Diagram of the unit cell for meta-objects, *p* = 180 μm, and *a* is 98 and 104 μm for R_A_ and R_B_, respectively. The bottom diagrams are two meta-objects for S_mb_ and S_mq_, which are combined with R_A_ and R_B_ in different orders. **c** Photo of the fabricated meta-object for imaging experiment. **d**, **g** PSNRs of the reconstructed images at different compression ratios for conventional CS and ACS algorithms. **e**, **h** Reconstructed images of S_mb_ and S_mq_ through 64 measurements with conventional CS and ACS algorithm at *f*_*1*_ and *f*_*2*_. **f**, **i** Pseudo-color images of S_mb_ and S_mq_ which are mixtures of the images at *f*_1_ and *f*_2_

We designed and fabricated artificially dispersive objects mixed with two different split-ring resonators (SRRs), i.e., R_A_ and R_B_, to verify the proposed algorithm for two-color CS imaging. Their periodicity is *p* = 180 μm, and the side length (*a*) is 98 and 104 μm for R_A_ and R_B_, respectively (refer to Fig. S4 for the test measurement). Fig. 3a shows that they are resonant at *f*_1_ and *f*_2_, respectively, and Fig. 3b shows the unit cell structures. We designed two imaging objects (S_mb_ and S_mq_) with R_A_ and R_B_ arranged in binary and quadrant types, as shown in Fig. 3b. The optical image of the fabricated sample is shown in Fig. 3c. Since the unit size of R_A_ and R_B_ is at the micron level, it is impossible to distinguish them with naked eyes.

Due to the remarkable contrast of spectra response for two different SRRs, the images of S_mb_ and S_mq_ will be different at *f*_1_ and *f*_2_. In the imaging experiments, we applied the Hadamard matrix with −1 and +1. However, it is well known that −1 cannot be directly obtained using an intensity-based modulator. To get the negative mask values for the two-color imaging, we collected data from two complementary masks with values of [1, 0] and subtracted them subsequently.

Since the peak signal-to-noise ratio (PSNR) is an important index to evaluate the quality of reconstructed images in CS imaging, we calculated it as follows:^44^

3 $${\mathrm{PSNR}} = 10 \times {{{\mathrm{log}}}}_{10}\left( {\frac{{(2^m - 1)^2}}{{{\mathrm{MSE}}}}} \right)$$

where *m* represents the bit-width of the pixel, the MSE (mean square error) represents the mean square error between reconstructed and actual imaging object data, which can be expressed as:

4 $${\mathrm{MSE}} = \frac{1}{n}\mathop {\sum}\limits_{j = 1}^n {\left\| {x_j - y_j} \right\|} ^2$$

where *n* represents the number of pixels of the image, *x*_*j*_ represents the pixel value of the actual object image, and *y*_*j*_ represents the pixel value of the corresponding reconstructed image. The PSNR is a metric of similarity, and the larger the value is, the higher the image similarity is.

After collecting the data, we first conducted image reconstruction of S_mb_ and S_mq_ at *f*_1_ using the conventional CS algorithm based on the L1 minimization algorithm, i.e., the nonuniformity of THz beam intensity distribution and the performance of pixels are not considered as priors. Though the transmittance difference between R_A_ and R_B_ in the samples is slight by only about 10%, our LC SLM can successfully reconstruct the images of object S_mb_ and S_mq_ at frequency *f*_1_.

To evaluate the effect of nonuniform beam shape and inhomogeneous MD across the pixels, we performed the reconstruction with ACS algorithm, which formulated these non-idealness factors into the forward imaging model of the conventional CS algorithm. By incorporating the sparsity prior to the target object, the smoothness prior to the THz source distribution, and the bound constraint upon the nonuniformity, the target could be reconstructed with elegant quality (please refer to the methods and supplementary section 7 for details). To demonstrate the effectiveness of the proposed method, we performed the conventional CS imaging and calculated the PSNR for comparison. Figure 3d, g reveal that, for both conventional CS and ACS algorithms, the image quality (or PSNR) improves with the increase of compression rate. The image qualities of S_mb_ and S_mq_ improve remarkably after using the ACS algorithm. For example, after taking 64 measurements, the ACS algorithm improves the image qualities of S_mb_ at *f*_1_ and *f*_2_ by 49% and 52%, respectively, compared with the CS algorithm. Meanwhile, the ACS algorithm only brings about 5.4 and 15% improvement in the image qualities of S_mq_ at *f*_1_ and *f*_2_, respectively, relative to the CS algorithm. From the perspective of the difference in sparseness, we restrict the sparseness in the gradient domain. There is a gradient at 16 pixels in the S_mq_ boundary region, so image quality improvement is not as high as S_mb_. The reconstructed images of S_mb_ and S_mq_ (Fig. 3e,h) were read out and represented as the blue and red false colors that were defined by the frequencies of *f*_1_ and *f*_2_, respectively. Then the colored images were mixed into the pseudo-color images, as shown in Fig. 3f, i. Similarly, the image qualities of pseudo-color images using the ACS algorithm are better than those using the conventional CS algorithm.

### CS imaging for nondispersive objects by frequency-switching

**Fig. 4:**
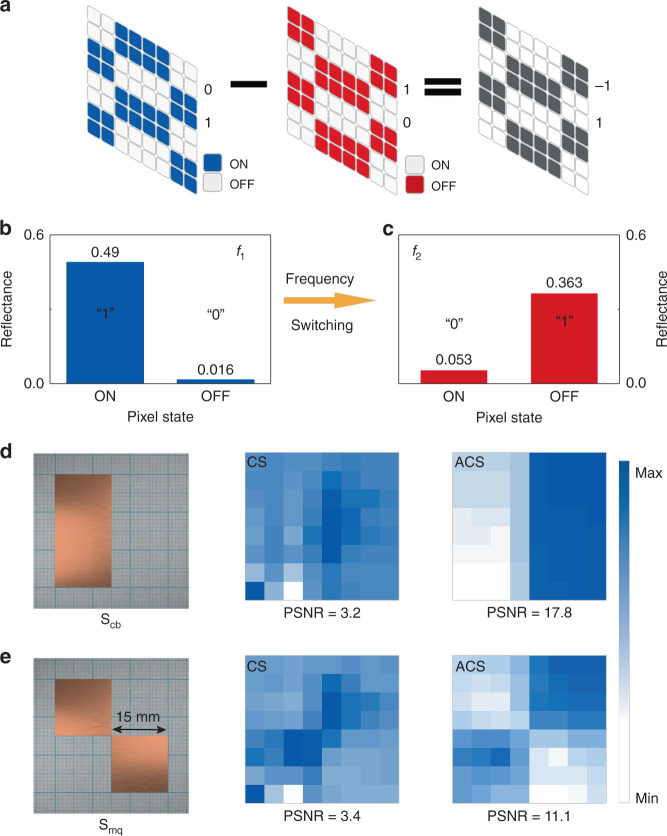
Frequency-switching method for CS imaging. **a** Schematic diagram for obtaining the Hadamard mask by frequency-switching. **b** Measured reflectance coefficient of SLM in ON and OFF states at *f*_1_. **c** Measured reflectance coefficient of SLM pixels in ON and OFF states at *f*_2_. **d**, **e** Reconstructed images of S_cb_ and S_cq_ with conventional CS and ACS algorithms

One feature of our LC SLM is its opposite MD at *f*_1_ and *f*_2_ (Fig. 2b). If the reflecting pixel in the high reflection state is defined as “1” and the pixel in the perfect absorption state is defined as “0”, when frequency switches to *f*_2_, the “1” state pixels at *f*_1_ will automatically change to “0” state pixels. and the “0” state at *f*_1_ flips to the “1” state, as shown in Fig. 4b, c. Correspondingly, the masks obtained at two frequencies are spatially complementary. Therefore, we can directly subtract the measurement masks at two frequencies to obtain the Hadamard mask, as illustrated in Fig. 4a. In other words, we can make a single Hadamard mask measurement by frequency-switching rather than changing the electric bias on each pixel. Compared with the sub-second scale switching time of LC under the electric field, the frequency-switching time of the THz source and a single data acquisition time is almost negligible. Therefore, the proposed method can significantly save imaging time.

To reconstruct the target object from the measurements at two different frequencies, we proposed an optimization algorithm for the frequency-switching CS imaging method. It takes the nonuniformity of the SLM and the THz source into account as well (see method and supplementary information for details). To verify the feasibility of the new method, we fabricated two imaging objects made from copper foil (copper binary sample S_cb_ and copper quadrant sample S_cq_), as shown in Fig. 4d,e. Likewise, we first used a conventional CS algorithm for reconstruction imaging on S_cb_ and S_cq_. The experimental results show that the imaging qualities are poor with PSNRs of about 3, and the objects are hardly distinguished. Given that the output power of the THz source at *f*_1_ and *f*_2_ is different, after the positive and negative mask subtraction operation, adverse effects such as the nonuniformity of pixel performance are doubled, leading to serious image blurs and low PSNRs. On the contrary, when the ACS algorithm is performed, the image quality is greatly improved. For S_cb_ and S_cq_, the PSNRs using the ACS algorithm are increased by 456% and 226% compared with the CS algorithm, respectively, as shown in Fig. 4d,e. Therefore, our ACS algorithm alone can elevate the frequency-switching CS imaging to a highly functional level even without extra workload such as multiple-output power calibration measurements.

## Discussion

The proposed dual-color THz LC SLM has shown advantages in improving the imaging speed and spectral imaging. The proposed frequency-switching method based on LC SLM and the ACS reconstruction algorithm could save nearly half of imaging time, showing vast potential for single-pixel imaging using frequency-selective SLMs, especially those based on LC, MEMS, and phase change materials^16,33,45^. In addition, the SLM with two or more operating frequencies can be used for imaging the dispersive objects, therefore providing a low-cost spectral imaging solution at THz frequencies. At present, most of the THz spectral imaging systems are based on the time-domain spectroscopic technique. Although the spectral resolution is high, it requires a high-power femtosecond laser, complicated optical path and components, and an expensive CCD camera^46^, limiting its popularity. The coherent receiver array is always costly and requires high local oscillator power. On the contrary, THz SLM with multiple working frequencies only requires a single THz broadband detector for spectral imaging. Thus, the system cost can be significantly reduced. Although the switching time of the proposed THz LC-SLM device is sub-second (see Fig. S6), there are still many possible avenues to improve the modulation speed, such as optimizing the structure of the unit cell, introducing ferroelectric liquid crystal or blue phase liquid crystal with faster response time^47,48^.

The major problems encountered in imaging applications are the nonuniform distribution of THz sources and the inhomogeneity of pixels. The proposed auto-calibration algorithm can effectively correct the errors and improve the imaging quality. To further validate our proposed method, we conducted computational experiments to reconstruct images with different nonuniformity of THz source intensity and pixel reflectance. Quantitative comparisons were also made with other common CS methods^49–51^ that are widely utilized in THz or other CS imaging experiments^11,20,22,24,52^ (see supplementary information section 7.4). Based on the experimental results, our proposed method provides an elegant solution to obtain high-fidelity images from imaging systems with different degrees of nonuniformity, thus lending more insight to THz CS imaging for further improvement of imaging quality. In this work, we only introduce the gradient domain sparsity prior to the target object and the smoothness prior to the THz source distribution to reduce the ill-posedness of the optimization problem and improve the reconstruction quality. With the development of deep learning, we can further introduce more sophisticated deep priors for the target objects in specific applications, such as security check and THz source distribution. The reconstruction effectiveness and efficiency will be further improved. Furthermore, training an end-to-end deep neural network that could automatically calibrate the effect of the THz source and nonuniform MD distribution will be a promising direction.

In conclusion, we developed an electrically programmable dual-color SLM based on LC. To reduce the effect of the nonuniform incidence beam and variational MD of pixels, we developed an ACS algorithm for image reconstruction in THz single-pixel imaging. We realized dual-color imaging of artificially constructed THz dispersive objects. When imaging objects without dispersion, the Hadamard mask can be obtained through rapid frequency-switching of the THz source, thus improving the imaging speed. This method provides a reliable and low-cost solution for single-pixel imaging. The proposed device has great potential for future applications such as projection display and single-pixel multispectral imaging.

## Materials and methods

### Device fabrication

The complementary patterns of the 8 × 8 pixelated patch array and periodic resonant structures are formed onto the bottom and top quartz substrates, respectively, using ultraviolet photolithography. The 200-nm-thick gold films were deposited onto the substrates using magnetron sputtering. Then, the lift-off process was used to form the metallic structures. Then, two gold bars were formed on the other side of the top quartz substrate, and they were electrically connected to the metallic resonator array layer with conductive silver glue. In the following, the bottom quartz substrate is glued to a pre-prepared PCB board. Two 10-μm-thick polyimide (PI) film stripes were attached to the two long sides of the lower quartz substrate, and the top quartz substrate was covered with the PI stripes. The AB glue was used to seal the surroundings of the PI film to form the LC box. Finally, the LC was instilled into the LC box after heating the LC to 106 °C.

### THz projection displays measurement

The THz continuous wave system was used for projection display measurement (see Fig. S5). The patterns of letters were loaded into the FPGA to control the state of each pixel. The SLM device was placed on a motorized positioning platform. The motorized positioning platform was used to control the focused THz spot scanning the entire surface of SLM. A THz zero-bias detector (ZBD) was used to collect the signal reflected by the SLM.

### ACS algorithm for dispersive objects

The transmission coefficient could be recovered in consideration of the THz source distribution (*s*) and modulation nonuniformity (*m*). At each frequency, the measurement matrix for positive and negative code is $M^ + = c^1M{{{\mathrm{{\Lambda}}}}}_{m^1} + c^0\left( {1 - M} \right){{{\mathrm{{\Lambda}}}}}_{m^0}\,{{{\mathrm{and}}}}\,{{{\mathrm{M}}}}^ - = c^1\left( {1 - M} \right){{{\mathrm{{\Lambda}}}}}_{m^1} + c^0M{{{\mathrm{{\Lambda}}}}}_{m^0}$, respectively. *c*^1^ and *c*^0^ denotes the measured average reflectance coefficient when all the pixels are turned on and off, respectively. Specifically, $c^1 = 0.49$ and 0.053 for *f*_1_ and *f*_2_, respectively. $c^0 = 0.016$ and 0.363 for *f*_1_ and *f*_2_, respectively. *m*^1^ and *m*^0^ denotes the nonuniformity when all the pixels are turned on and off, respectively. The practical measurement matrix corresponding to each Hadamard mask is $M = M^ + - M^ - = \left( {2M - 1} \right)\left( {c^1{{{\mathrm{{\Lambda}}}}}_{m^1} - c^0{{{\mathrm{{\Lambda}}}}}_{m^0}} \right) = \left( {2M - 1} \right){{{\mathrm{{\Lambda}}}}}_m$. Denoting the Hadamard mask Φ = 2*M*−1, the optimization problem in consideration of the nonuniformity factors can be formulated as:

5 $$\min \lambda _s\left\{ {\left\| {\nabla _xs} \right\|_2^2 + \left\| {\nabla _ys} \right\|_2^2} \right\} + \lambda _g\left\{ {\left\| {\nabla _xI} \right\|_1 + \left\| {\nabla _yI} \right\|_1} \right\} + \mu \left\| {{\Phi}{{{\mathrm{{\Lambda}}}}}_m{{{\mathrm{{\Lambda}}}}}_sI - y} \right\|_2^2 + \mu _0\left\| {{\Phi}{{{\mathrm{{\Lambda}}}}}_ms - y_0} \right\|_2^2 s.t.\left\| m \right\| \le r$$

where Λ_*_ is the diagonalized matrix of vector *, *s* is the intensity distribution of the THz source, *y*_0_ and *y* is the measurement without and with the target. The measurement without any object (*y*_0_) is pre-captured. *I* denotes the transmission coefficient of the unknown target; *m* is the nonuniform scaling factor of the SLM; *r* represents the upper bound of the nonuniform scaling factor. The first term of the objective function denotes the smoothness prior to the imposed THz source and non-homogeneity of pixels, and the second term represents the spatial sparsity prior set upon the reconstructed target object. The third and fourth terms denote the data term corresponding to the measurement of the target object and the pre-captured data with no target object, respectively. We introduce the upper bound constraint upon the nonuniformity scaling factor *m* to further constrain the optimization. By solving this optimization problem with the alternating direction of multipliers^53^, we could obtain the transmission distribution coefficients of the target object. The nonuniformity factors of ${\Lambda}_m$ and ${\Lambda}_s$ are also reconstructed, which helps to alleviate or even eliminate the effect of nonuniformity.

### ACS algorithm for nondispersive objects by frequency-switching

For simplification, here we use notifications $M_i^ + = M{{{\mathrm{{\Lambda}}}}}_{m_i^1}c_i^1 + \left( {1 - M} \right){{{\mathrm{{\Lambda}}}}}_{m_i^0}c_i^0,\,M_i^ - = \left( {1 - M} \right){{{\mathrm{{\Lambda}}}}}_{m_i^1}c_i^1 + M{{{\mathrm{{\Lambda}}}}}_{m_i^0}c_i^0,\,(i = 1,2)$, where $m_i^1$ and $m_i^0$ denotes the non-idealness from the nonuniform distribution of pixel reflectance when all the pixels are turned on or off, respectively. To reconstruct the target object considering the THz source distribution and nonuniformity modulation, we pre-capture the measurement with no target object of modulation matrix *M* and 1 – *M* at *f*_1_ and *f*_2_, *i*.*e*., *y*0_1_^+^, *y*0_1_^−^, *y*0_2_^+^, *y*0_2_^−^. For each target, we capture two sets of measurements with the modulation matrix *M* at *f*_1_ and *f*_2_, i.e., *y*_1_^+^ and *y*_2_^+^. To reconstruct the target object from the measurement, we solve the following optimization problem:

6 $$\begin{array}{ll}&\min \lambda _g\left\{ {\left\| {\nabla _xI} \right\|_1 + \left\| {\nabla _yI} \right\|_1} \right\} + \mathop {\sum}\limits_{i = 1,2} {\lambda _{s_i}\left\{ {\left\| {\nabla _xs_i} \right\|_2^2 + \left\| {\nabla _ys_i} \right\|_2^2} \right\}} \\ &\quad+\, \mathop {\sum}\limits_{i = 1,2} {\mu _i\left\{ {\left\| {M_i^ + {{{\mathrm{{\Lambda}}}}}_{s_i}I - y_i^ + } \right\|_2^2} \right\}} + \mathop {\sum}\limits_{i = 1,2} {\mu 0_i^ + \left\{ {\left\| {M_i^ + s_i - y0_i^ + } \right\|_2^2} \right\}} \\ &\quad+ \,\mathop {\sum}\limits_{i = 1,2} {\mu 0_i^ - \left\{ {\left\| {M_i^ - s_i - y0_i^ - } \right\|_2^2} \right\}} \\&\qquad s.t.\,\left| {m_{1/2}^{0/1}} \right| \le r\end{array}$$

using the alternating direction of multipliers^53^, where *s*_*i*_ denotes the nonuniform THz source intensity distribution at the frequency of *f*_1_. Through reconstructing the objective transmission and the nonuniform factors of $m_i^1$, $m_i^0$, and *s*_*i*_, the deteriorate effect caused by these nonuniform factors could be removed and the imaging results could thus be auto-calibrated. The optimization derivation details are provided in the supplementary information.

## Supplementary information

Supplementary Information for Dual-color THz spatial light modulator for single-pixel imaging
