# Supplementary material for: Dual-color terahertz spatial light modulator for single-pixel imaging
Source: Light Sci Appl. 2022 Jun 23;11:191. doi: 10.1038/s41377-022-00879-5 (PMC9225988; doi:10.1038/s41377-022-00879-5)
Supplement: Supplementary file 1 — Supplementary Information for Dual-color THz spatial light modulator for single-pixel imaging [file 41377_2022_879_MOESM1_ESM.docx]

Supplementary Information for

**Dual-color terahertz spatial light modulator for single-pixel imaging**

Weili Li^1^, Xuemei Hu^2^, Jingbo Wu^1,3,*^, Kebin Fan^1,3,*^, Benwen Chen^1^, Caihong Zhang^1,3^, Wei Hu^4^, Xun Cao^2^, Biaobing Jin^1,3,*^, Yanqing Lu^4^, Jian Chen^1,3^, Peiheng Wu^1^

^1^Research Institute of Superconductor Electronics (RISE), School of Electronic Science and Engineering, Nanjing University, Nanjing 210023, China

^2^ School of Electronic Science and Engineering, Nanjing University, Nanjing 210023, China

^3^Purple Mountain Laboratories, Nanjing 211111, China

^4^National Laboratory of Solid State Microstructures, Collaborative Innovation Center of Advanced Microstructures and College of Engineering and Applied Sciences, Nanjing University, 163 Xianlin Avenue, Nanjing 210023, China

*jbwu@nju.edu.cn, kebin.fan@nju.edu.cn, bbjin@nju.edu.cn

In this supplementary document, we provide the characterization of dual-frequency liquid crystal, the metasurface absorber design, the response time of proposed SLM, the fabrication process of meta-object, the projection display measurement, the experimental setup for single-pixel imaging, and auto-calibrated CS algorithm.

**1. Characterization of dual-frequency liquid crystal**

We selected the dual-frequency liquid crystal (DP002-016) in our design because it has large birefringence, and the alignment layer is not required to define the initial orientation of liquid crystal molecules. We measured the dual-frequency liquid crystal using the THz time-domain spectroscopy (TDS) system (THz photonics TPF15K) shown in Fig. S1.


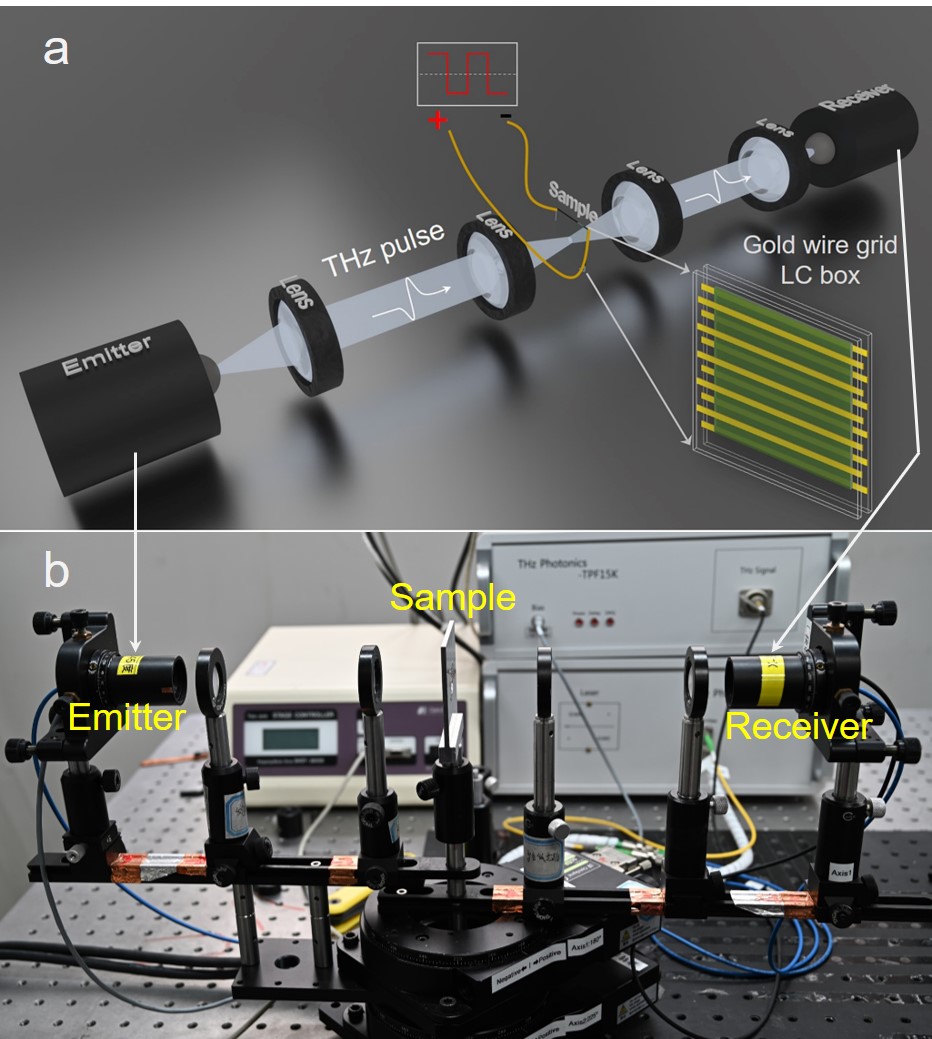


**Fig. S1** Schematic diagram **a** and photo **b** of the THz-TDS system for liquid crystal characterization.

For the characterization of the birefringence of liquid crystal, two gold wire-grid polarizers with a line width of 10 μm and spacing of 10 μm were fabricated onto two 300 μm-thick quartz substrates, respectively. Then the two substrates were bonded together to form a 140 μm-thick liquid crystal box, and the double-frequency liquid crystal was infiltrated into the box. A 140 μm-thick blank liquid crystal box was fabricated for reference. The transmission spectra of the liquid crystal box were measured by applying square wave voltage bias with different amplitudes between the metallic wires on the top and bottom substrates. The measured refractive index (*n*) is defined as:

 (S1)

where *Δϕ* represents the phase delay difference between the filled and blank liquid crystal box, *d* represents the thickness of the liquid crystal layer. The measured refractive indexes of liquid crystal with a bias of 20 V square wave voltage and without bias are shown in Fig. S2. A birefringence of 0.17 is achieved by switching the electric bias in the frequency range of 400 and 500 GHz.


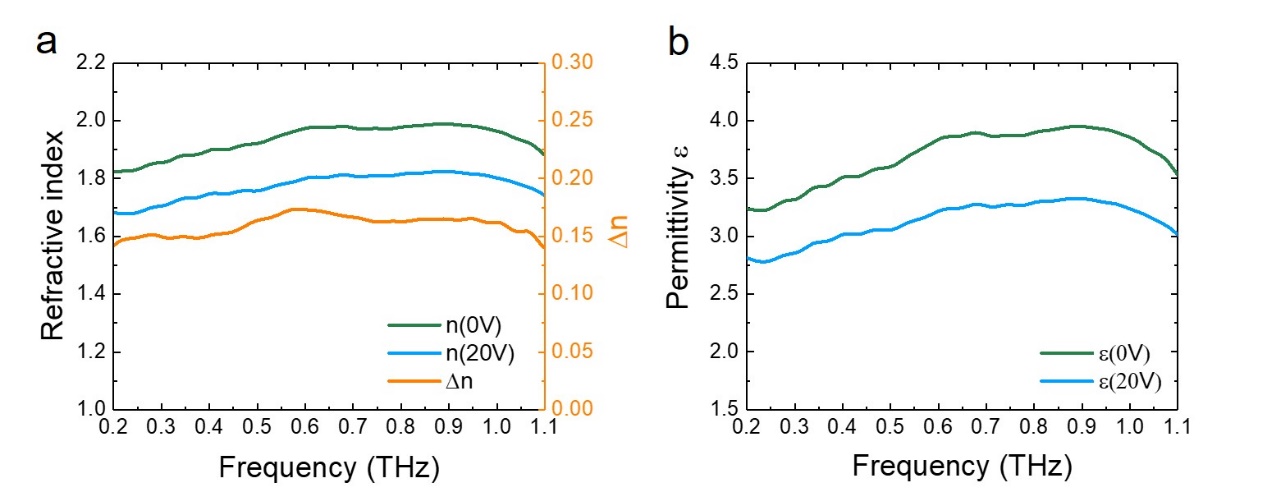


**Fig. S2** Measured refractive index **a** and permittivity **b** at different frequencies.

**2 Metasurface absorber theory and design**

Metasurface absorbers (MMA) in our design are composed of metallic resonators, dielectric, and metallic ground planes, as shown in Fig. S3a. The resonators at the top layer produce the electric response that determines the permittivity. Meanwhile, the antiparallel current in the top resonators and the ground plane determines the magnetic responses and the permeability as shown in Fig.3b. By modifying the geometry of the absorber, the electric and magnetic responses can be adjusted. When the impedance at the resonant frequency is designed to be equal to the vacuum impedance, and the imaginary part of the refractive index is enormous, there is a significant absorption to incident THz wave. After geometry optimization based on the simulation results, a practical design of perfect absorption at the resonant frequency is obtained.

The temporal coupled-mode theory (TCMT) can be used to clarify the working mechanism of the metamaterial absorber[^1^](#_ENREF_1). As shown in Fig. S3a, there are three decay channels to dissipate the incoming energy, including the radiative decay rate γ_rad_, the nonradiative decay rates of γ_LC_ and γ_Au_ from the intrinsic material loss of materials. The total absorption near the resonance can then be expressed as follows[^2^](#_ENREF_2)^,^[^3^](#_ENREF_3):

$A(\omega)=\frac{4\gamma_{\text{rad}}\gamma_{0}}{(\omega-\omega_{0})^{2}+(\gamma_{\text{rad}}+\gamma_{0})^{2}}$ (S2)

where, γ_0_ = γ_LC_ +γ_Au_ is the total nonradiative loss rate. According to reference [2] and [3], γ_rad_, γ_Au,_ and γ_LC_ are all related to the thickness of the liquid crystal layer. We will obtain an optimized thickness that the radiative loss is equal to the sum of the nonradiative losses, *i*.*e*., γ_rad_ = γ_LC_ +γ_Au_. In that case, the absorption rate *A*(ω) = 1. We simulated the absorption spectra of different thicknesses as shown in Fig. S3c. When the thickness of the liquid crystal layer is 10 μm, the perfect absorption is achieved.


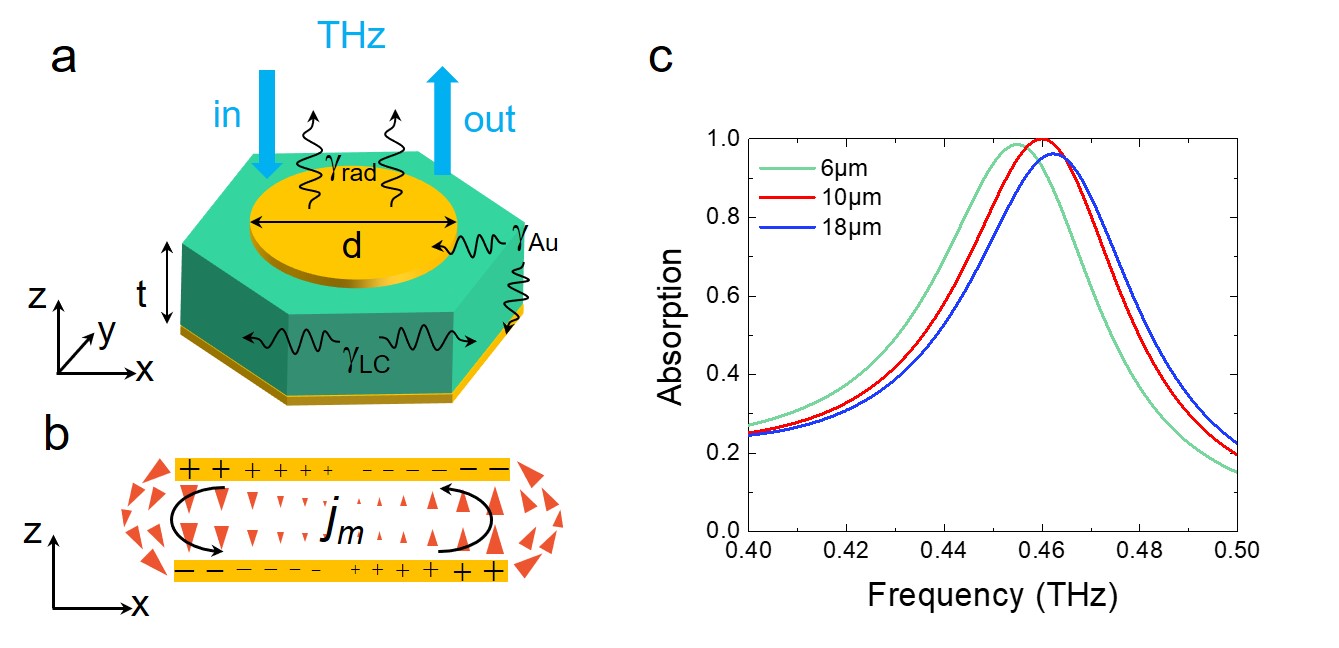


**Fig. S3** **a** Diagram of metasurface absorber. **b** Charge distribution, electric field, and magnetic loop current are generated by the metamaterial absorber at the resonant frequency. **c** Absorption spectra of metasurface absorbers with different LC layer thicknesses (*t*).

**3. Fabrication process and measurement of meta-object**

To verify the capability of SLM in single-pixel imaging of dispersive objects, we need the dispersive materials with significantly different transmission coefficients at *f*_1_ and *f*_2_. We designed metamaterials consisting of periodic split-ring resonators (SRRs). The fundamental mode resonance frequency of SRRs can be adjusted by changing the side length (*a*). We simulated the transmission spectra of SRRs with *a* ranging from 97 to 105 μm, as shown in Fig. S4a.

To characterize the performance of the SRR, we fabricated the SRR arrays with various *a* on the 300 μm-thick quartz substrates as shown in Fig. S4b. The transmission spectra shown in Fig. S4b are smoothed curves of the measurement data. Fig. S4c shows the experimental setup for the THz transmission spectra measurement. In the system, the samples are placed at THz focus plane, and the transmission spectra were obtained by sweeping frequency. Due to the deviation of between geometry parameters of the fabricated sample and the simulation ones, there are slight differences in the transmission spectra as shown in Fig. S4a and Fig. S4b. Based on the experimental results, SRRs with *a* = 98 μm and 104 μm were selected as the meta-object elements because their resonance frequencies are close to *f*_1_ and *f*_2_, respectively.


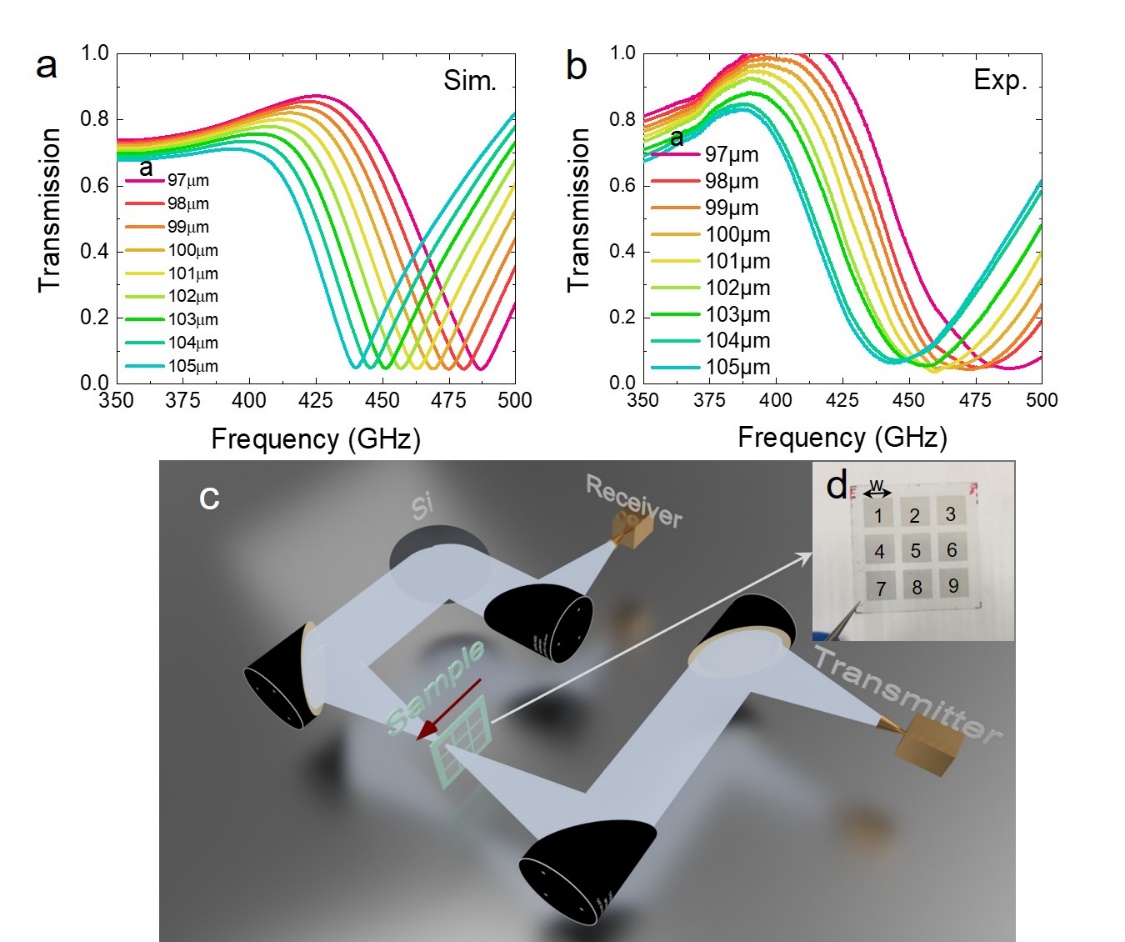


**Fig. S4** **a** Simulated transmission spectra of the SRR with different *a*. **b** Measured transmission spectra of the SRR with various *a* after smoothing. **c** Diagram of the experimental setup for THz transmission spectra measurement. **d** Photo of fabricated sample of SRR array.

**4 Projection display measurement**

The experimental setup for projection display measurement is shown in Fig. S5. The THz beam is focused onto the SLM device. The device is fixed on the motorized positioning platform, which controls the movement of the device in a two-dimensional plane. Thus, the reflection coefficient distribution on the SLM surface can be mapped out. The THz waves reflected by SLM are received by a single THz detector after passing through a beam splitter (BS) and OAPM. In our measurement, the size of the THz light spot is larger than the total area of a single pixel in SLM. The motorized positioning stage moves 0.5 mm at each step.


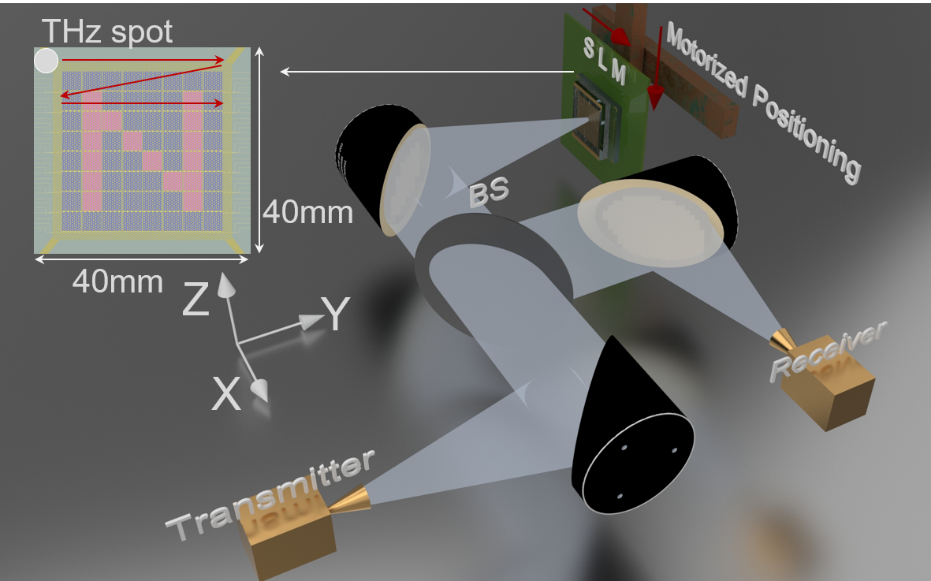


**Fig. S5** Schematic of the experimental setup for projection display measurement.

**5 Response time of proposed SLM**

**Fig. S6** SLM response time measurment

The response time is one of the key devices performance indicators. Using the experimental setup shown in Fig. S5, we measured the response time of the SLM by switching all the pixels between ON and OFF states. As shown in Fig. S6, the response time is about 400 ms when the amplitude changes from 10% to 90% or vice versa[^4^](#_ENREF_4).

**6. Experimental setup for single-pixel imaging**

The experimental setup for single-pixel imaging is shown in Fig. S7. The THz beam generated from the source was collimated by an off-axis parabolic mirror (OAPM). The THz collimation beam passes through the imaging object and is then modulated by the SLM. The measurement matrix was loaded into the FPGA, and the SLM was controlled by it to generate the corresponding mask patterns. The spatial modulated THz beam was focused and collected by a single THz detector. For conventional compressive sensing (CS) measurement, we interchanged the 0 and 1 in the measurement matrix and repeated the measurement without altering source frequency. After subtracting the measurement data, the measurement data based on the Hadamard matrix is obtained and used for image reconstruction. For the frequency switching CS, if the measurement matrix consisting of 0 and 1 is loaded into the FPGA, the mask patterns generated from SLM are complementary. Therefore, the measurement data based on the Hadamard matrix was obtained by subtracting the measurement data at *f*_1_ and *f*_2_.


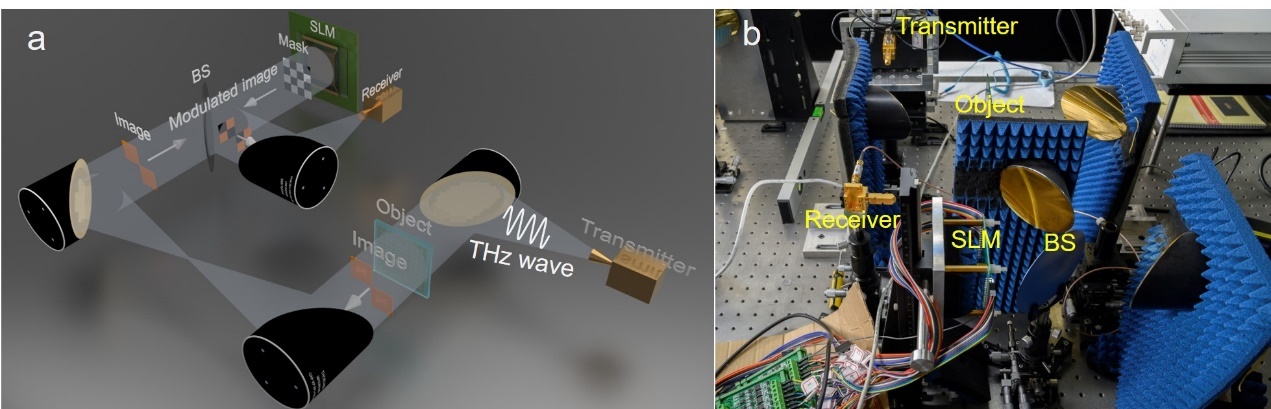


**Fig. S7** Schematic diagram **a** and photo **b** of the THz continuous-wave system for single-pixel imaging.

**7 Auto-calibrated CS algorithm**

The nonuniformity of beam intensity distribution and pixel performance are the main factors that cause the poor quality of conventional CS imaging. To inhomogeneity of light intensity and pixel performance, we propose the auto-calibrated CS algorithm. The details of the algorithm are discussed below.

**7.1 Optimization algorithm for dispersive target object:**

Under each frequency, the measurement matrix for positive code is $M^{+}=c^{1}M\Lambda_{m^{1}}+c^{0}\left( 1-M \right)\Lambda_{m^{0}}, M^{-}=c^{1}\left( 1-M \right)\Lambda_{m^{1}}+c^{0}M\Lambda_{m^{0}},$ $M=M^{+}-M^{-}=\left( 2M-1 \right)\left( {c^{1}\Lambda}_{m^{1}}-{c^{0}\Lambda}_{m^{0}} \right)=(2M-1)\Lambda_{m}$, denoting $\Phi=2M-1,$ the optimization problem is then

$\begin{matrix} \min\lambda_{s}\left\{\left\|\nabla_{x}s\right\|_{2}^{2}+\left\|\nabla_{y}s\right\|_{2}^{2}\right\}+\lambda_{g}\left\{\left\|\nabla_{x}I\right\|_{1}+\left\|\nabla_{y}I\right\|_{1}\right\}+\mu\left\|\Phi\Lambda_{m}\Lambda_{s}I-y\right\|_{2}^{2} \\ +\mu_{0}\left\|\Phi\Lambda_{m}s-y_{0}\right\|_{2}^{2} \\ s.t.\left\|m\right\|\leq r \end{matrix}$ (S3)

With variable substitution ${z_{x}=\nabla}_{x}I$*,* ${z_{y}=\nabla}_{y}I$, the objective function could be rewritten as:

$\begin{matrix} \min\lambda_{s}\left\{\left\|\nabla_{x}s\right\|_{2}^{2}+\left\|\nabla_{y}s\right\|_{2}^{2}\right\}+\lambda_{g}\left\{\left\|z_{x}\right\|_{1}+\left\|z_{y}\right\|_{1}\right\}+\mu\left\|\Phi\Lambda_{m}\Lambda_{s}I-y\right\|_{2}^{2} \\ +\mu_{0}\left\|\Phi\Lambda_{m}s-y_{0}\right\|_{2}^{2} \\ s.t.\left\{\begin{matrix} \nabla_{x}I=z_{x} \\ \nabla_{y}I=z_{y} \\ \left\|m\right\|\leq r \end{matrix}\right. \end{matrix}$ (S4)

With augmented Lagrangian method[^5^](#_ENREF_5):

$\begin{matrix} f(I,s,m,z,v)=\lambda_{s}\left\{\left\|\nabla_{x}s\right\|_{2}^{2}+\left\|\nabla_{y}s\right\|_{2}^{2}\right\}+\lambda_{g}\left\{\left\|z_{x}\right\|_{1}+\left\|z_{y}\right\|_{1}\right\} \\ +\mu\left\|\Phi\Lambda_{m}\Lambda_{s}I-y\right\|_{2}^{2}+\mu_{0}\left\|\Phi\Lambda_{m}s-y_{0}\right\|_{2}^{2}+\sum_{j=x,y} \frac{\rho}{2}\left\|\nabla_{j}I-z_{j}+\frac{1}{\rho}v_{j}\right\|_{2}^{2} \\ s.t.\left\|m\right\|\leq r \end{matrix}$ (S5)

With the alternating direction of multiplier algorithm[^6^](#_ENREF_6), the method could be divided into multiple sub-optimization problems, and the optimization algorithm is then:

$\begin{matrix} I^{k+1}:=\mathrm{argmin}_{I}\left(f(I,s_{i}^{k},m_{i}^{*k},v_{j}^{k},z_{j}^{k})\right) \\ s_{i}^{k+1}:=\mathrm{argmin}_{s_{i}}\left(f(I^{k+1},s_{i},m_{i}^{*k},v_{j}^{k},z_{j}^{k})\right) \\ m_{i}^{*k+1}:=\mathrm{argmin}m_{i}^{*}\left(f(I^{k+1},s_{i}^{k+1},m_{i}^{*},v_{j}^{k},z_{j}^{k})\right) \\ z_{j}^{k+1}:=\mathrm{argmin}z_{j}\left(f(I^{k+1},s_{i}^{k+1},m_{i}^{*k+1},v_{j}^{k},z_{j}^{k})\right) \\ v_{j}^{k+1}:=v_{j}^{k}+\rho(\nabla_{j}I^{k+1}-z_{j}^{k+1}) \end{matrix}$ (S6)

1. For optimizing I, the other variable is fixed, and the objective function with respect to I is:

$f(I)=\mu\left\|\Phi\Lambda_{m}\Lambda_{s}I-y\right\|_{2}^{2}+\sum_{j=x,y} \frac{\rho}{2}\left\|\nabla_{j}I-z_{j}+\frac{1}{\rho}v_{j}\right\|_{2}^{2}$ (S7)

$\frac{\partial f(I)}{\partial I}=2\mu(\Phi\Lambda_{m}\Lambda_{s})^{T}(\Phi\Lambda_{m}\Lambda_{s}I-y)+\sum_{j=x,y} \rho\nabla_{j}^{T}(\nabla_{j}I-z_{j}+\frac{1}{\rho}v_{j})$ (S8)

$\begin{matrix} I^{k+1}=\left[2\mu(\Phi\Lambda_{m^{k}}\Lambda_{s^{k}})^{T}\Phi\Lambda_{m^{k}}\Lambda_{s^{k}}+\sum_{j=x,y} \rho\nabla_{j}^{T}\nabla_{j}\right]^{-1} \\ \cdot\left[2\mu(\Phi\Lambda_{m^{k}}\Lambda_{s^{k}})^{T}y+\sum_{j=x,y} \rho\nabla_{j}^{T}(z_{j}^{k}-\frac{1}{\rho}v_{j}^{k})\right] \end{matrix}$ (S9)

1. For optimizing s, the other variable is fixed, and the objective function with respect to *s* is:

$f(s)=\lambda_{s}\left\{\left\|\nabla_{x}s\right\|_{2}^{2}+\left\|\nabla_{y}s\right\|_{2}^{2}\right\}+\mu\left\|\Phi\Lambda_{m}\Lambda_{I}s-y\right\|_{2}^{2}+\mu_{0}\left\|\Phi\Lambda_{m}s-y_{0}\right\|_{2}^{2}$ (S10)

$\begin{matrix} \frac{\partial f(s)}{\partial s}=2\lambda_{s}(\nabla_{x}^{T}\nabla_{x}s+\nabla_{y}^{T}\nabla_{y}s)+2\mu(\Phi\Lambda_{m}\Lambda_{I})^{T}(\Phi\Lambda_{m}\Lambda_{I}s-y) \\ +2\mu_{0}(\Phi\Lambda_{m})^{T}(\Phi\Lambda_{m}s-y_{0}) \end{matrix}$ (S11)

$\begin{matrix} s^{k+1}=\left[2\lambda_{s}(\nabla_{x}^{T}\nabla_{x}+\nabla_{y}^{T}\nabla_{y})+2\mu(\Phi\Lambda_{m^{k}}\Lambda_{I^{k+1}})+2\mu_{0}(\Phi\Lambda_{m^{k}})^{T}\Phi\Lambda_{m^{k}}\right]^{-1} \\ \cdot\left[2\mu(\Phi\Lambda_{m^{k}}\Lambda_{I^{k+1}})^{T}y+2\mu_{0}(\Phi\Lambda_{m^{k}})^{T}y_{0}\right] \end{matrix}$ (S12)

1. For optimizing *m*, the other variable is fixed, and the objective function with respect to *m* is:

$f(m)=\mu\left\|\Phi\Lambda_{s}\Lambda_{I}m-y\right\|_{2}^{2}+\mu_{0}\left\|\Phi\Lambda_{s}m-y_{0}\right\|_{2}^{2}$ (S13)

$\frac{\partial f(m)}{\partial m}=2\mu(\Phi\Lambda_{s}\Lambda_{I})^{T}(\Phi\Lambda_{s}\Lambda_{I}m-y)+2\mu_{0}(\Phi\Lambda_{s})^{T}(\Phi\Lambda_{s}m-y_{0})$ (S14)

$\begin{matrix} m^{k+1}=\left[2\mu(\Phi\Lambda_{s^{k+1}}\Lambda_{I^{k+1}})^{T}\Phi\Lambda_{s^{k+1}}\Lambda_{I^{k+1}}+2\mu_{0}(\Phi\Lambda_{s^{k+1}})^{T}\Phi\Lambda_{s^{k+1}}\right]^{-1} \\ \cdot\left[2\mu(\Phi\Lambda_{s^{k+1}}\Lambda_{I^{k+1}})^{T}y+2\mu_{0}(\Phi\Lambda_{s^{k+1}})^{T}y_{0}\right] \end{matrix}$ (S15)

1. Hard projecting *m* to the bound r, corresponding to the constraint:

$m=\text{project}(m,r)=\left\{\begin{matrix} r,\text{ if }x>r \\ x,\text{ if abs}(x)\leq r \\ -r,\text{ if }x<-r \end{matrix}\right.$ (S16)

1. For optimizing $z_{j}$, the other variable is fixed, and the objective function with respect to $z_{j}$ is:

$f(z_{j})=\lambda_{g}\left\|z_{j}\right\|_{1}+\frac{\rho}{2}\left\|\nabla_{j}I-z_{j}+\frac{1}{\rho}v_{j}\right\|_{2}^{2}$ (S17)

$z_{j}^{k+1}=\text{shrinkage(}\nabla_{\text{j}}I\text{+}\frac{1}{\rho}\text{v}_{\text{j}}\text{,}\frac{\lambda_{g}}{\rho}\text{)}$ (S18)

$\text{shrinkage(}x,r\text{)=}\left\{\begin{matrix} x-r,\text{ if }x>r \\ 0,\text{ if abs(}x\text{)}\leq\text{r} \\ x+r,\text{ if }x<-r \end{matrix}\right.$ (S19)

1. Update conjugate multiplier

$v_{j}^{k+1}:=v_{j}^{k}+\rho(\nabla_{j}I^{k+1}-z_{j}^{k+1})$ (S20)

1. Here we choose the penalty parameter updating rule:

$\begin{matrix} \mu=\text{min(}\mu\text{*a,}\mu_{\text{max}}\text{)} \\ \mu_{0}=\min(\mu_{0}*a,\mu_{\text{max}}) \end{matrix}$ (S21)

1. Empirically chosen hyperparameters: $r=1.2c^{1}, \mu=\mu_{0}=\rho=0.1, \lambda_{s}=\lambda_{I}=1, \lambda_{g}=5, a = 1.05, \mu_{max}=1e3$, respectively.


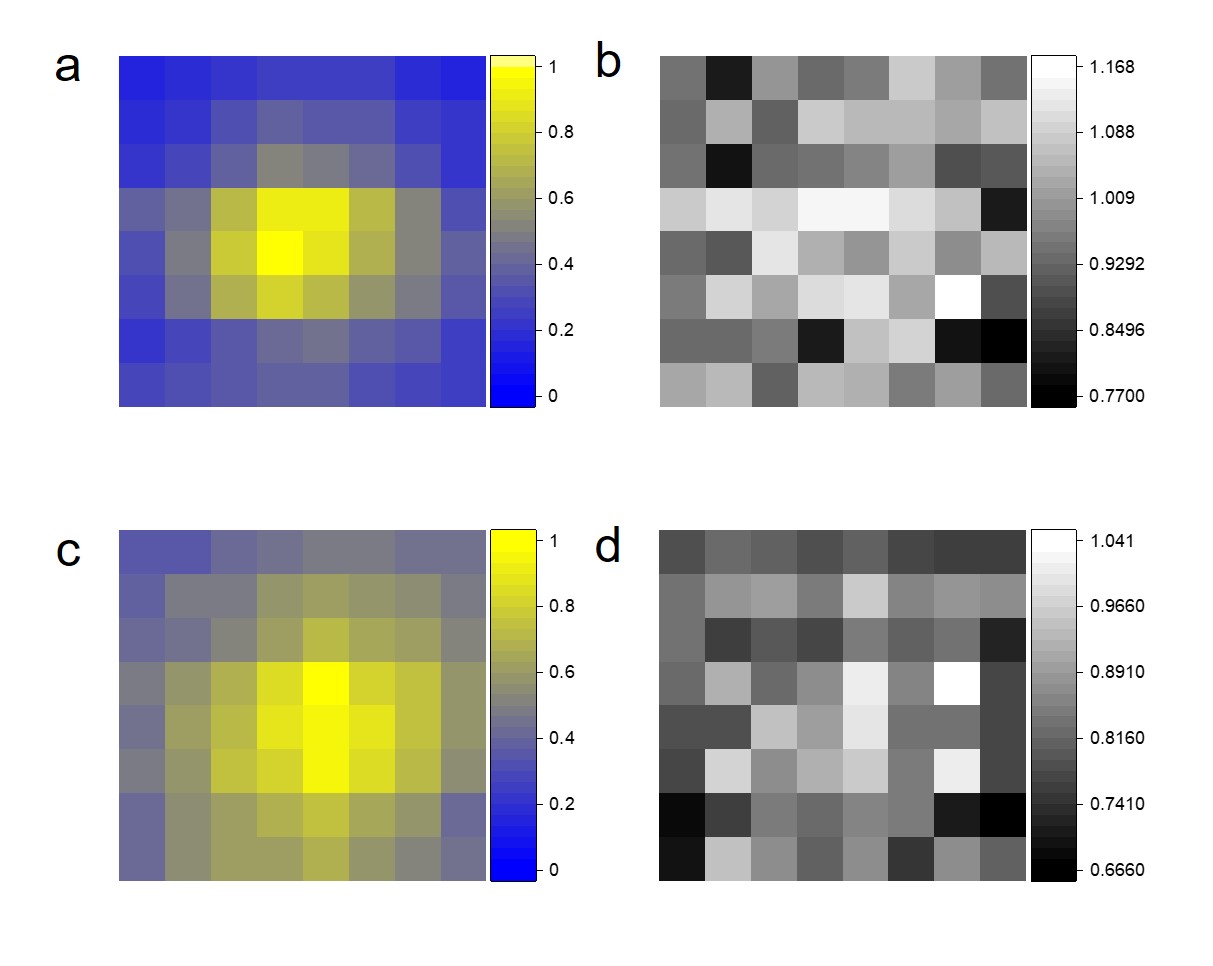


**Fig. S8** **a** THz source distribution and **b** the nonuniform performance of each pixel at *f*_1_, **c** THz source distribution, and **d** the nonuniform performance at *f*_2_.

The THz source and nonuniform distributions at frequency *f*_1_ and *f*_2_ could be estimated with the proposed algorithm. The obtained results are shown in Fig. S8.

**7.2 Optimization problem for nondispersive object**

For simplification, here we use notifications $M_{i}^{+}=M\Lambda_{m_{i}^{1}}c_{i}^{1}+\left( 1-M \right)\Lambda_{m_{i}^{0}}c_{i}^{0}, M_{i}^{-}=\left( 1-M \right)\Lambda_{m_{i}^{1}}c_{i}^{1}+M\Lambda_{m_{i}^{0}}c_{i}^{0}, (i=1,2)$.

The objective function of our method can be represented as:

$\begin{matrix} \min\lambda_{g}\left\{\left\|\nabla_{x}I\right\|_{1}+\left\|\nabla_{y}I\right\|_{1}\right\}+\sum_{i=1,2} \lambda_{s_{i}}\left\{\left\|\nabla_{x}s_{i}\right\|_{2}^{2}+\left\|\nabla_{y}s_{i}\right\|_{2}^{2}\right\}+\sum_{i=1,2} \mu_{i}\left\{\left\|M_{i}^{+}\Lambda_{s_{i}}I-y_{i}^{+}\right\|_{2}^{2}\right\} \\ +\sum_{i=1,2} \mu0_{i}^{+}\left\{\left\|M_{i}^{+}s_{i}-y0_{i}^{+}\right\|_{2}^{2}\right\}+\sum_{i=1,2} \mu0_{i}^{-}\left\{\left\|M_{i}^{-}s_{_{i}}-y0_{i}^{-}\right\|_{2}^{2}\right\} \\ s.t.\left|m_{1/2}^{0/1}\right|\leq r \end{matrix}$ (S22)

With variable substitution ${z_{x}=\nabla}_{x}I$*,* ${z_{y}=\nabla}_{y}I$, the objective function could be rewritten as:

 (S23)

With augmented Lagrangian method[^5^](#_ENREF_5):

$\begin{matrix} f(I,s_{*},m_{*}^{*})=\lambda_{g}\sum_{j=x,y} \left\|z_{j}\right\|_{1}+\sum_{i=1,2} \lambda_{s_{i}}\left\{\left\|\nabla_{x}s_{i}\right\|_{2}^{2}+\left\|\nabla_{y}s_{i}\right\|_{2}^{2}\right\}+\sum_{i=1,2} \mu_{i}\left\{\left\|M_{i}^{+}\Lambda_{s_{i}}I-y_{i}^{+}\right\|_{2}^{2}\right\} \\ +\sum_{i=1,2} \mu0_{i}^{+}\left\{\left\|M_{i}^{+}s_{i}-y0_{i}^{+}\right\|_{2}^{2}\right\}+\sum_{i=1,2} \mu0_{i}^{-}\left\{\left\|M_{i}^{-}s_{i}-y0_{i}^{+}\right\|_{2}^{2}\right\} \\ \sum_{j=x,y} \frac{\rho}{2}\left\|\nabla_{j}I-z_{j}+\frac{1}{\rho}v_{j}\right\|_{2}^{2} \\ s.t\left|m_{1/2}^{0/1}\right|\leq r \end{matrix}$ (S24)

With alternating direction of multipliers[^6^](#_ENREF_6), the method could be divided into multiple sub-optimization problems, and the optimization algorithm is then:

$\begin{matrix} I^{k+1}:=\text{argmin}_{\text{I}}(f(I,s_{i}^{k},m_{i}^{*k},v_{j}^{k},z_{j}^{k})) \\ s_{i}^{k+1}:=\text{argmin}_{s_{i}}(f(I^{k+1},s_{i},m_{i}^{*k},v_{j}^{k},z_{j}^{k})) \\ m_{i}^{*k+1}:=\text{argmin}_{m_{i}^{*}}(f(I^{k+1},s_{i}^{k+1},m_{i}^{*},v_{j}^{k},z_{j}^{k})) \\ z_{j}^{k+1}:=\text{argmin}_{z_{j}}(f(I^{k+1},s_{i}^{k+1},m_{i}^{*k+1},v_{j}^{k},z_{j}^{k})) \\ v_{j}^{k+1}:=v_{j}^{k}+\rho(\nabla_{j}I^{k+1}-z_{j}^{k+1}) \end{matrix}$ (S25)

1. For optimizing I, the other variable is fixed and the objective function with respect to I is:

$f(I)=\sum_{i=1,2} \mu_{i}\left\{\left\|M_{i}^{+}\Lambda_{s_{i}}I-y_{i}^{+}\right\|_{2}^{2}\right\}+\sum_{j=x,y} \frac{\rho}{2}\left\|\nabla_{j}I-z_{j}+\frac{1}{\rho}v_{j}\right\|_{2}^{2}$ (S26)

$\frac{\partial f(I)}{\partial I}=\sum_{i=1,2} 2\mu_{i}(M_{i}^{+}\Lambda_{s_{i}})^{T}(M_{i}^{+}\Lambda_{s_{i}}I-y_{i}^{+})+\sum_{j=x,y} \rho\nabla_{j}^{T}(\nabla_{j}I-z_{j}+\frac{1}{\rho}v_{j})=0$ (S27)

$\begin{matrix} I^{k+1}=\left[\sum_{i=1,2} 2\mu_{i}(M_{i}^{+}\Lambda_{s_{i}})^{T}(M_{i}^{+}\Lambda_{s_{i}})+\sum_{j=x,y} \rho\nabla_{j}^{T}\nabla_{j}\right]^{-1} \\ \cdot\left[\sum_{i=1,2} 2\mu_{i}(M_{i}^{+}\Lambda_{s_{i}})^{T}y_{i}^{+}+\sum_{j=x,y} \rho\nabla_{j}^{T}(z_{j}-\frac{1}{\rho}v_{j})\right] \end{matrix}$ (S28)

1. For optimizing $s_{i}$, the other variable is fixed and the objective function with respect to $s_{i}$ is:

$\begin{matrix} f(s_{i})=\lambda_{s_{i}}\left\{\left\|\nabla_{x}s_{i}\right\|_{2}^{2}+\left\|\nabla_{y}s_{i}\right\|_{2}^{2}\right\}+\mu_{i}\left\{\left\|M_{i}^{+}\Lambda_{I^{k+1}}s_{i}-y_{i}^{+}\right\|_{2}^{2}\right\} \\ +\mu0_{i}^{+}\left\{\left\|M_{i}^{+}s_{i}-y0_{i}^{+}\right\|_{2}^{2}\right\}+\mu0_{i}^{-}\left\{\left\|M_{i}^{-}s_{i}-y0_{i}^{-}\right\|_{2}^{2}\right\} \end{matrix}$ (S29)

$\begin{matrix} \frac{\partial f(s_{i})}{\partial s_{i}}=2\lambda_{s_{i}}(\nabla_{x}^{T}\nabla_{x}s_{i}+\nabla_{y}^{T}\nabla_{y}s_{i})+2\mu_{i}(M_{i}^{+}\Lambda_{I^{k+1}})^{T}(M_{i}^{+}\Lambda_{I^{k+1}}s_{i}-y_{i}^{+}) \\ 2\mu0_{i}^{+}(M_{i}^{+})^{T}(M_{i}^{+}s_{i}-y0_{i}^{+})+2\mu0_{i}^{-}(M_{i}^{-})^{T}(M_{i}^{-}s_{i}-y0_{i}^{-})=0 \end{matrix}$ (S30)

$\begin{matrix} s_{i}^{k+1}=\left[\begin{matrix} \lambda_{s_{i}}(\nabla_{x}^{T}\nabla_{x}+\nabla_{y}^{T}\nabla_{y})+\mu_{i}(M_{i}^{+}\Lambda_{I^{k+1}})^{T}M_{i}^{+}\Lambda_{I^{k+1}}+\mu0_{i}^{+}(M_{i}^{+})^{T}M_{i}^{+} \\ +\mu0_{i}^{-}(M_{i}^{-})^{T}M_{i}^{-} \end{matrix}\right]^{-1} \\ \cdot\left[\mu_{i}(M_{i}^{+}\Lambda_{I^{k+1}})^{T}y_{i}^{+}+\mu0_{i}^{+}(M_{i}^{+})^{T}y0_{i}^{+}+\mu0_{i}^{-}(M_{i}^{-})^{T}y0_{i}^{-}\right] \end{matrix}$ (S31)

1. For optimizing $m_{i}^{1}$, the other variable is fixed, and the objective function with respect to $m_{i}^{1}$ is:

$\begin{matrix} f(m_{i}^{1})=\sum_{i=1,2} \mu_{i}\left\{\left\|\left[c_{i}^{1}M\Lambda_{m_{i}^{1}}+c_{i}^{0}(1-M)\Lambda_{m_{i}^{0}}\right]\Lambda_{s_{i}}I-y_{i}^{+}\right\|_{2}^{2}\right\} \\ +\sum_{i=1,2} \mu0_{i}^{+}\left\{\left\|\left[c_{i}^{1}M\Lambda_{m_{i}^{1}}+c_{i}^{0}(1-M)\Lambda_{m_{i}^{0}}\right]s_{i}-y0_{i}^{+}\right\|_{2}^{2}\right\} \\ +\sum_{i=1,2} \mu0_{i}^{-}\left\{\left\|\left[c_{i}^{1}(1-M)\Lambda_{m_{i}^{1}}+c_{i}^{0}M\Lambda_{m_{i}^{0}}\right]s_{i}-y0_{i}^{-}\right\|_{2}^{2}\right\} \end{matrix}$ (S32)

Which can be rewritten as:

$\begin{matrix} f(m_{i}^{1})=\mu_{i}\left\|c_{i}^{1}M\Lambda_{s_{i}^{k+1}}\Lambda_{I^{k+1}}m_{i}^{1}+c_{i}^{0}(1-M)\Lambda_{(m_{i}^{0})^{k}}\Lambda_{s_{i}^{k+1}}I^{k+1}-y_{i}^{+}\right\|_{2}^{2} \\ +\mu0_{i}^{+}\left\|c_{i}^{1}M\Lambda_{s_{i}^{k+1}}m_{i}^{1}+c_{i}^{0}(1-M)\Lambda_{(m_{i}^{0})^{k}}s_{i}^{k+1}-y0_{i}^{+}\right\|_{2}^{2} \\ +\mu0_{i}^{-}\left\|c_{i}^{1}(1-M)\Lambda_{s_{i}^{k+1}}m_{i}^{1}+c_{i}^{0}M\Lambda_{(m_{i}^{0})^{k}}s_{i}^{k+1}-y0_{i}^{-}\right\|_{2}^{2} \end{matrix}$ (S33)

$\begin{matrix} \frac{\partial f(m_{i}^{1})}{\partial m_{i}^{1}}=2\mu_{i}(c_{i}^{1}M\Lambda_{s_{i}^{k+1}}\Lambda_{I^{k+1}})^{T}\left[\begin{matrix} c_{i}^{1}M\Lambda_{s_{i}^{k+1}}\Lambda_{I^{k+1}}m_{i}^{1}+ \\ c_{i}^{0}(1-M)\Lambda_{(m_{i}^{0})^{k}}\Lambda s_{i}^{k+1}I^{k+1}-y_{i}^{+} \end{matrix}\right] \\ +2\mu0_{i}^{+}(c_{i}^{1}M\Lambda_{s_{i}^{k+1}})^{T}\left[c_{i}^{1}M\Lambda_{s_{i}^{k+1}}m_{i}^{1}+c_{i}^{0}(1-M)\Lambda_{(m_{i}^{0})^{^{k}}}s_{i}^{k+1}-y0_{i}^{+}\right] \\ +2\mu0_{i}^{-}\left[c_{i}^{1}(1-M)\Lambda_{s_{i}^{k+1}}\right]^{T}\left[c_{i}^{1}(1-M)\Lambda_{s_{i}^{k+1}}m_{i}^{1}+c_{i}^{0}M\Lambda_{(m_{i}^{0})^{^{k}}}s_{i}^{k+1}-y0_{i}^{-}\right] \end{matrix}$ (S34)

$\begin{matrix} (m_{i}^{1})^{k+1}=\left\{\begin{matrix} \mu_{i}(c_{i}^{1}M\Lambda_{s_{i}^{k+1}}\Lambda_{I^{k+1}})^{T}c_{i}^{1}M\Lambda_{s_{i}^{k+1}}\Lambda_{I^{k+1}}+\mu0_{i}^{+}(c_{i}^{1}M\Lambda_{s_{i}^{k+1}})^{T}c_{i}^{1}M\Lambda_{s_{i}^{k+1}} \\ +\mu0_{i}^{-}\left[c_{i}^{1}(1-M)\Lambda_{s_{i}^{k+1}}\right]^{T}c_{i}^{1}(1-M)\Lambda_{s_{i}^{k+1}} \end{matrix}\right\}^{-1} \\ \cdot\left\{\begin{matrix} \mu_{i}(c_{i}^{1}M\Lambda_{s_{i}^{k+1}}\Lambda_{I^{k+1}})^{T}\left[y_{i}^{+}-c_{i}^{0}(1-M)\Lambda_{(m_{i}^{0})^{k}}\Lambda_{s_{i}^{k+1}}I^{k+1}\right] \\ +\mu0_{i}^{+}(c_{i}^{1}M\Lambda_{s_{i}^{k+1}})^{T}\left[y0_{i}^{+}-c_{i}^{0}(1-M)\Lambda_{(m_{i}^{0})^{k}}s_{i}^{k+1}\right] \\ +\mu0_{i}^{-}\left[c_{i}^{1}(1-M)\Lambda_{s_{i}^{k+1}}\right]^{T}\left[y0_{i}^{-}-c_{i}^{0}M\Lambda_{(m_{i}^{0})^{k}}s_{i}^{k+1}\right] \end{matrix}\right\} \end{matrix}$ (S35)

1. For optimizing $m_{i}^{0}$, the other variable is fixed, and the objective function with respect to $m_{i}^{1}$ is:

$\begin{matrix} f(m_{i}^{0})=\mu_{i}\left\|\left[c_{i}^{1}M\Lambda_{m_{i}^{1}}+c_{i}^{0}(1-M)\Lambda_{m_{i}^{0}}\right]\Lambda_{s_{i}}I-y_{i}^{+}\right\|_{2}^{2} \\ +\mu0_{i}^{+}\left\|\left[c_{i}^{1}M\Lambda_{m_{i}^{1}}+c_{i}^{0}(1-M)\Lambda_{m_{i}^{0}}\right]s_{i}-y0_{i}^{+}\right\|_{2}^{2} \\ +\mu0_{i}^{-}\left\|\left[c_{i}^{1}(1-M)\Lambda_{m_{i}^{1}}+c_{i}^{0}M\Lambda_{m_{i}^{0}}\right]s_{i}-y0_{i}^{+}\right\|_{2}^{2} \end{matrix}$ (S36)

Which can be rewritten as:

$\begin{matrix} f(m_{i}^{0})=\mu_{i}\left\|c_{i}^{1}M\Lambda_{(m_{i}^{1})^{k+1}}\Lambda_{s_{i}^{k+1}}I^{k+1}+c_{i}^{0}(1-M)\Lambda_{I^{k+1}}\Lambda_{s_{i}^{k+1}}m_{i}^{0}-y_{i}^{+}\right\|_{2}^{2} \\ +\mu0_{i}^{+}\left\|c_{i}^{1}M\Lambda_{(m_{i}^{1})^{k+1}}s_{i}^{k+1}+c_{i}^{0}(1-M)\Lambda_{s_{i}^{k+1}}m_{i}^{0}-y0_{i}^{+}\right\|_{2}^{2} \\ +\mu0_{i}^{-}\left\|c_{i}^{1}(1-M)\Lambda_{(m_{i}^{1})^{k+1}}s_{i}^{k+1}+c_{i}^{0}M\Lambda_{s_{i}^{k+1}}m_{i}^{0}-y0_{i}^{-}\right\|_{2}^{2} \end{matrix}$ (S37)

$\begin{matrix} \frac{\partial f(m_{i}^{0})}{\partial m_{i}^{0}}=2\mu_{i}\left(c_{i}^{0}(1-M)\Lambda_{I^{k+1}}\Lambda_{s_{i}^{k+1}}\right)^{T} \\ \cdot\left[c_{i}^{1}M\Lambda_{(m_{i}^{1})^{k+1}}\Lambda_{s_{i}^{k+1}}I^{k+1}+c_{i}^{0}(1-M)\Lambda_{I^{k+1}}\Lambda_{s_{i}^{k+1}}m_{i}^{0}-y_{i}^{+}\right] \\ +2\mu0_{i}^{+}\left(c_{i}^{0}(1-M)\Lambda_{s_{i}^{k+1}}\right)^{T}\left[c_{i}^{1}M\Lambda_{(m_{i}^{1})^{k+1}}s_{i}^{k+1}+c_{i}^{0}(1-M)\Lambda_{s_{i}^{k+1}}m_{i}^{0}-y0_{i}^{+}\right] \\ +2\mu0_{i}^{-}\left[c_{i}^{0}M\Lambda_{s_{i}^{k+1}}\right]^{T}\left[c_{i}^{1}(1-M)\Lambda_{(m_{i}^{1})^{k+1}}s_{i}^{k+1}+c_{i}^{0}M\Lambda_{s_{i}^{k+1}}m_{i}^{0}-y0_{i}^{-}\right] \end{matrix}$ (S38)

$\begin{matrix} (m_{i}^{0})^{k+1}=\left\{\begin{matrix} \mu_{i}\left(c_{i}^{0}(1-M)\Lambda_{I^{k+1}}\Lambda_{s_{i}^{k+1}}\right)^{T}c_{i}^{0}(1-M)\Lambda_{I^{k+1}}\Lambda_{s_{i}^{k+1}} \\ +\mu0_{i}^{+}\left(c_{i}^{0}(1-M)\Lambda_{s_{i}^{k+1}}\right)^{T}c_{i}^{0}(1-M)\Lambda_{s_{i}^{k+1}} \\ +\mu0_{i}^{-}\left[c_{i}^{0}M\Lambda_{s_{i}^{k+1}}\right]^{T}c_{i}^{0}M\Lambda_{s_{i}^{k+1}} \end{matrix}\right\}^{\text{-1}} \\ \cdot\left\{\begin{matrix} \mu_{i}\left(c_{i}^{0}(1-M)\Lambda_{I^{k+1}}\Lambda_{s_{i}^{k+1}}\right)^{T}\left[y_{i}^{+}-c_{i}^{1}M\Lambda_{(m_{i}^{1})^{k+1}}\Lambda_{s_{i}^{k+1}}I^{k+1}\right] \\ +\mu0_{i}^{+}\left(c_{i}^{0}(1-M)\Lambda_{s_{i}^{k+1}}\right)^{T}\left[y0_{i}^{+}-c_{i}^{1}M\Lambda_{(m_{i}^{1})^{k+1}}s_{i}^{k+1}\right] \\ +\mu0_{i}^{-}\left[c_{i}^{0}M\Lambda_{s_{i}^{k+1}}\right]^{T}\left[y0_{i}^{-}-c_{i}^{1}(1-M)\Lambda_{(m_{i}^{1})^{k+1}}s_{i}^{k+1}\right] \end{matrix}\right\} \end{matrix}$ (S39)

1. The hard projecting *m* to the bound *r*, corresponding to the constraint:

$m_{i}^{*}=\text{project(}m_{i}^{*},r\text{)=}\left\{\begin{matrix} r,\text{ if }x>r \\ x,\text{ if abs(}x\text{)}\leq\text{r} \\ -r,\text{ if }x<-r \end{matrix}\right.$ (S40)

1. For optimizing $z_{j}$, the other variable is fixed, and the objective function with respect to $z_{j}$ is:

$f(z_{j})=\lambda_{g}\left\|z_{j}\right\|_{1}+\frac{\rho}{2}\left\|\nabla_{j}I-z_{j}+\frac{1}{\rho}v_{j}\right\|_{2}^{2}$ (S41)

$z_{j}^{k+1}=\text{shrinkage(}\nabla_{j}I+\frac{1}{\rho}v_{j}\text{,}\frac{\lambda_{g}}{\rho}\text{)}$ (S42)

$\text{shrinkage(}x,r\text{)=}\left\{\begin{matrix} x-r,\text{ if }x>r \\ 0,\text{ if abs(}x\text{)}\leq\text{r} \\ x+r,\text{ if }x<-r \end{matrix}\right.$ (S43)

1. Update conjugate multiplier:

$v_{j}^{k+1}:=v_{j}^{k}+\rho(\nabla_{j}I^{k+1}-z_{j}^{k+1})$ (S44)

1. Here we choose the penalty parameter updating rule:

$\mu_{*}^{*}=\min(\mu_{*}^{*}*a,\mu_{\max})$ (S45)

1. Empirically chosen hyperparameters: $r=1.2, \mu_{i}=\mu0_{i}=\mu_{i}^{+}={\mu0}_{i}^{+}=\rho=0.1, \lambda_{s}=1, \lambda_{g}=5, a = 1.05, \mu_{max}=1e3$.

The estimated results of $m_{1}^{+}, m_{1}^{-},m_{2}^{+},m_{2}^{-}$ are shown in Fig. S9.


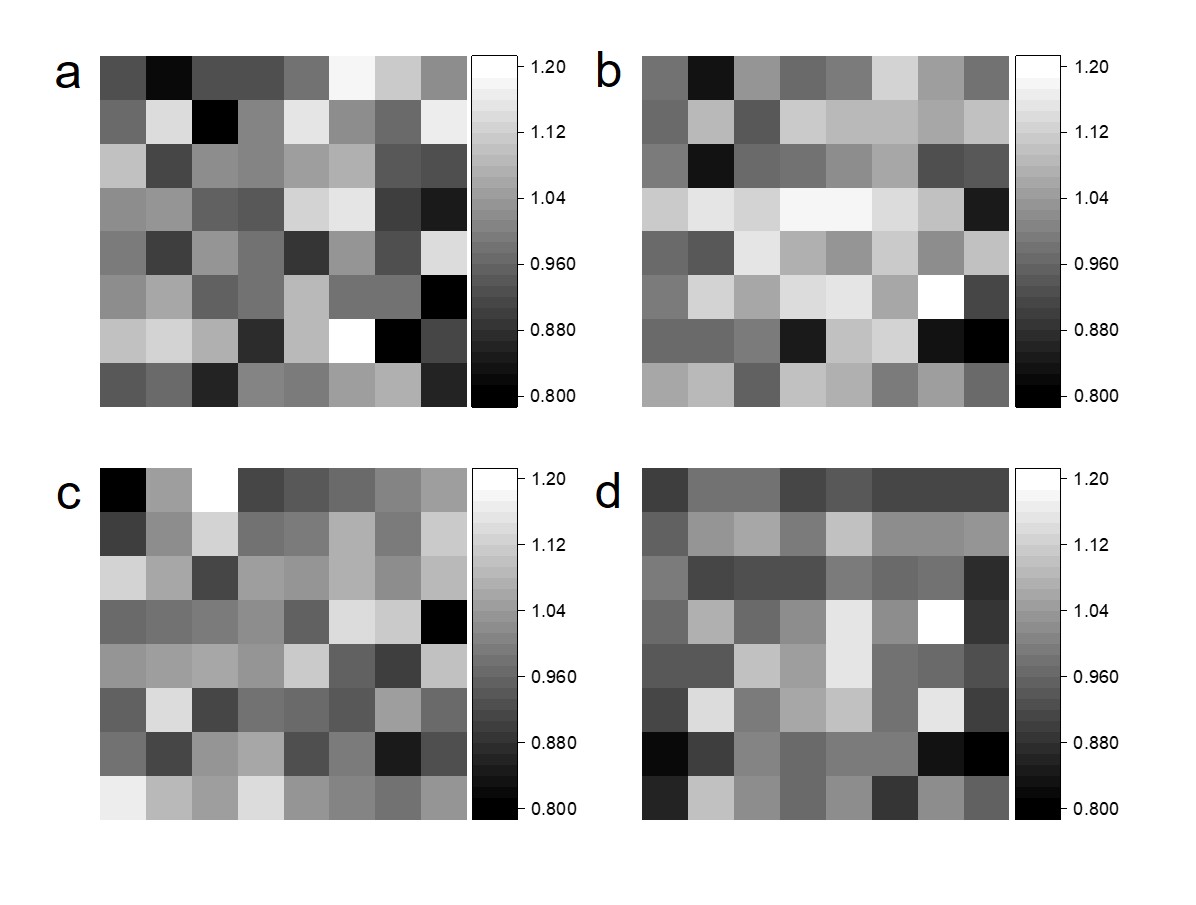
**Fig. S9** Estimated nonuniformity distribution, **a** $m_{1}^{+}$ **b** $m_{1}^{-}$ **c** $m_{2}^{+}$ and **d** $m_{2}^{-}$.

**7.3 Computational efficiency**

We compare these algorithms with the same computing environment, *i.e.*, Mac OSX system with 2.3 GHz Intel Core i7 processor and 32 GB CPU memory. The computing time comparison is shown in Table 1. Since our algorithm introduces reconstructing the distribution of the object and the nonuniform related factors, the convergence speed is 4-5 times slower than the conventional methods. However, the benefit is that the reconstruction performance is highly improved, as shown in Fig. 3 and Fig. 4. Furthermore, our algorithm is not specifically optimized for speed. By introducing accelerating techniques such as multi-core or multi-threading-based parallel computing techniques, the proposed algorithm could be vastly accelerated and facilitate real-time reconstruction.

|  | Conventional CS algorithm | Algorithm for dispersive object | Algorithm for non-dispersive object |
| --- | --- | --- | --- |
| Computing time | 0.04 s | 0.178 s | 0.183 s |

**Table. 1** Computing efficiency comparison.

**7.4** **Performances analysis with other methods in THz related single-imaging system.**

To thoroughly demonstrate the advantage of the proposed method, we compare it with compressive sensing (CS) imaging reconstruction algorithms^49-51^, which are widely adopted in THz or other CS imaging experiments, such as fast iterative shrinkage-thresholding algorithm (FISTA)^24, 11^, total variation (TV) regularized CS imaging^20, 22^ and alternating projection (AP) method^52^. We simulate nonuniform light beams with different Gaussian widths and nonuniform reflectance of spatial light modulators with different nonuniform scales. Since the existing methods are designed for CS imaging in the single-wavelength interrogation scenarios, we compare with existing methods^49-51^, *i.e.*, FISTA^49^, TV^50^, AP^51^ with the proposed optimization problems in Eq. 5. To avoid the effect of outliers, we utilize the binary objects adopted in the main article (Fig. 3 in the main article) and 50 randomly sampled images from the BSD300 dataset^52^ as the test image dataset. Here, for consistency, we randomly crop the image size to 32$\times$32. The PSNR is averaged over the test image dataset to compare the performance of different methods.

As mentioned in the manuscript, there are two main types of nonuniformity in the experiments, *i.e.*, the inhomogeneity of the THz source distribution and the nonuniform reflectance on the spatial light modulator. To verify our proposed methods with more experiments, we simulate the forward imaging process with different nonuniform degrees of the THz source intensity and the pixel reflectance.

**Nonuniform THz source**: The Gaussian shape of THz source intensity obeys the following intensity distribution:

$G\left( x \right)=\frac{1}{\sqrt{2\pi}\sigma}e^{\frac{\left( x-x_{c} \right)^{2}+\left( y-y_{c} \right)^{2}}{2{\pi\sigma}^{2}}}$ (S46)

where *σ* denotes the standard deviation of the Gaussian distribution, which could indicate the size of the Gaussian beam. We simulate similar experiments with different *σ*. As shown in Fig. S10, the nonuniformity of the THz source intensity decreases with the increase of $\sigma$. When $\sigma\geq40$, the nonuniformity caused by the THz source could be neglected. With these different intensity distributions of THz source, we could analyze the performance of different algorithms with varying degrees of nonuniformity.


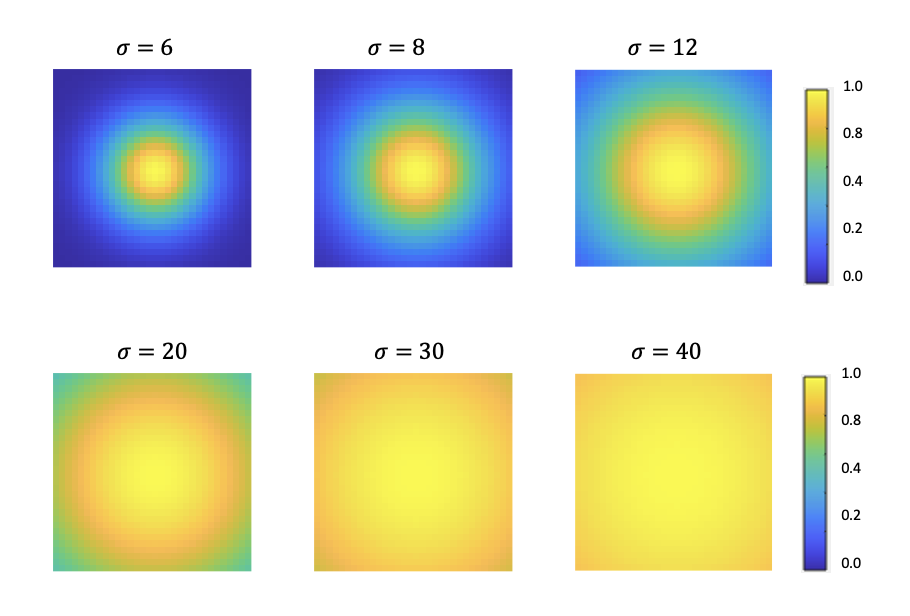


**Fig. S10** Simulated THz source intensity distributions with different *σ*.

**Nonuniform pixel reflectance:** To further investigate the effect of the nonuniformity of the pixel reflectance, we simulate the nonuniformity with varying *Λ_m_*, which quantifies the degree of nonuniformity in pixel reflectance. Here we simulate the reflectance map as a random distribution with different nonuniformity scales defined as

$r_{ns}=\frac{|\boldsymbol{m}_{max}-\boldsymbol{m}_{min}|}{\bar{m}}$ (S47)

where *m* denotes the diagonal elements of *Λ_m_* and $\bar{m}$ denotes the mean of *m*. We simulate $m$ with six different degrees of *r_ns_* to verify the proposed auto-correlation algorithm. The simulated maps of pixel reflectance with different *r_ns_* are shown in Fig. S11.


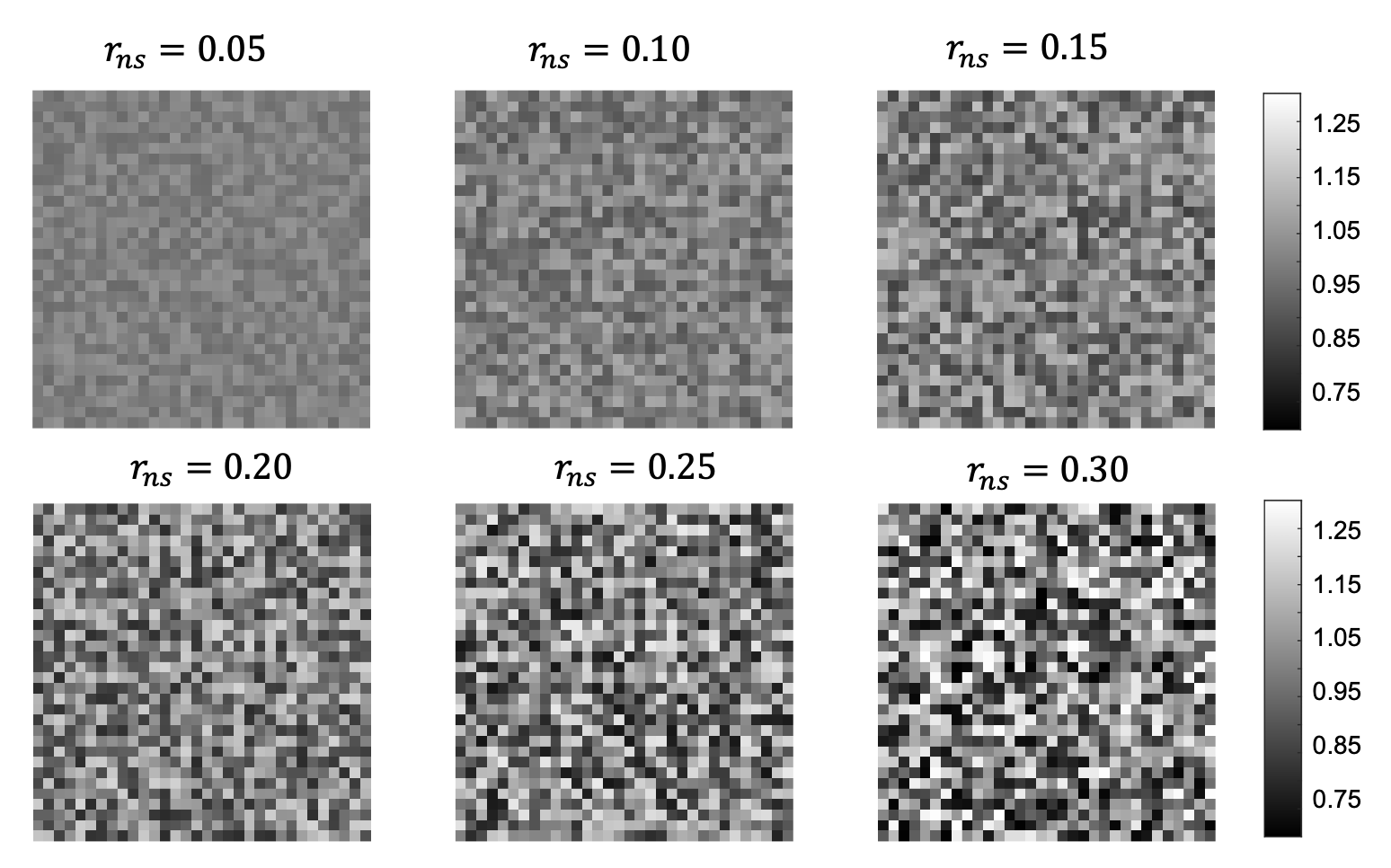


**Fig. S11** Simulated maps of pixel reflectance with different *r_ns_*


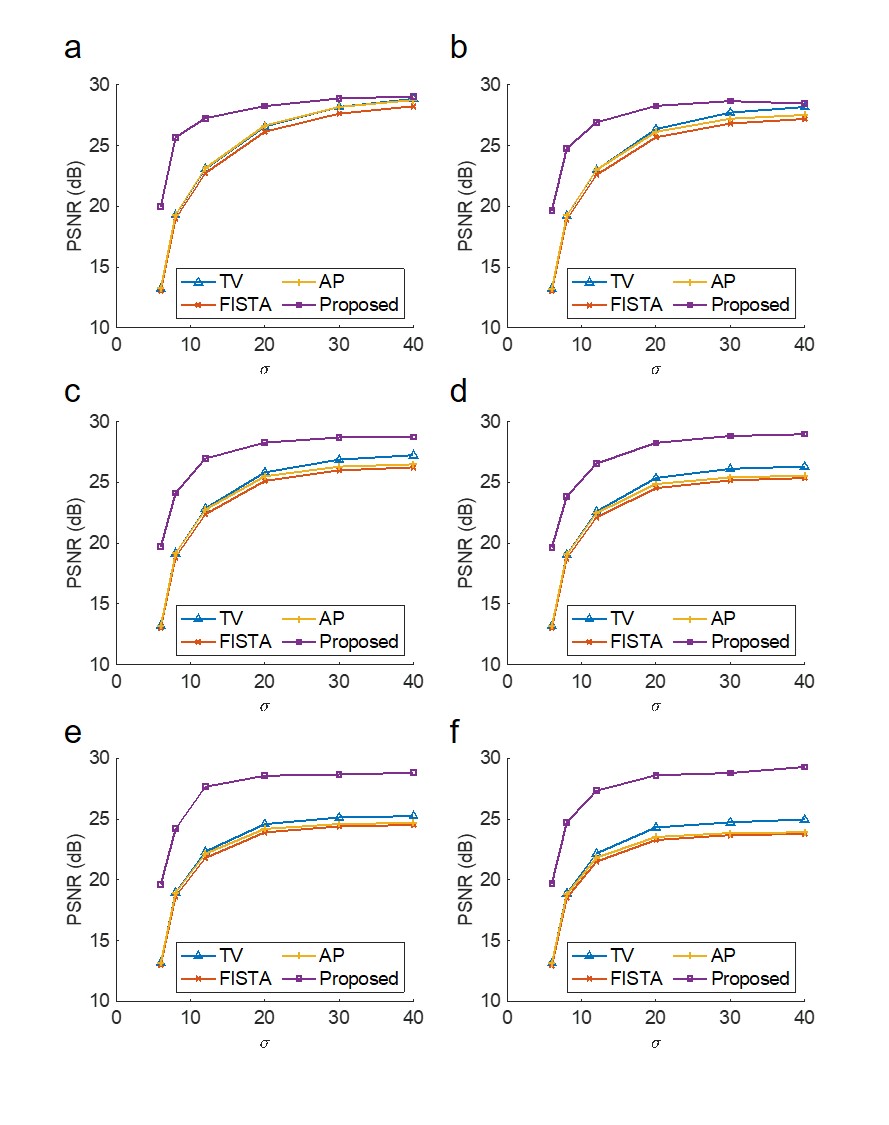


**Fig. S12** Calculated PSNR as a function of *σ* with *r_ns_* = 0.05 **a** *r_ns_* = 0.10 **b** *r_ns_* = 0.15 **c** *r_ns_* = 0.20 **d** *r_ns_* = 0.25 **e** and *r_ns_* = 0.30 **f** for different methods including FISTA^49^, TV^50^ and AP^51^ and our proposed method.


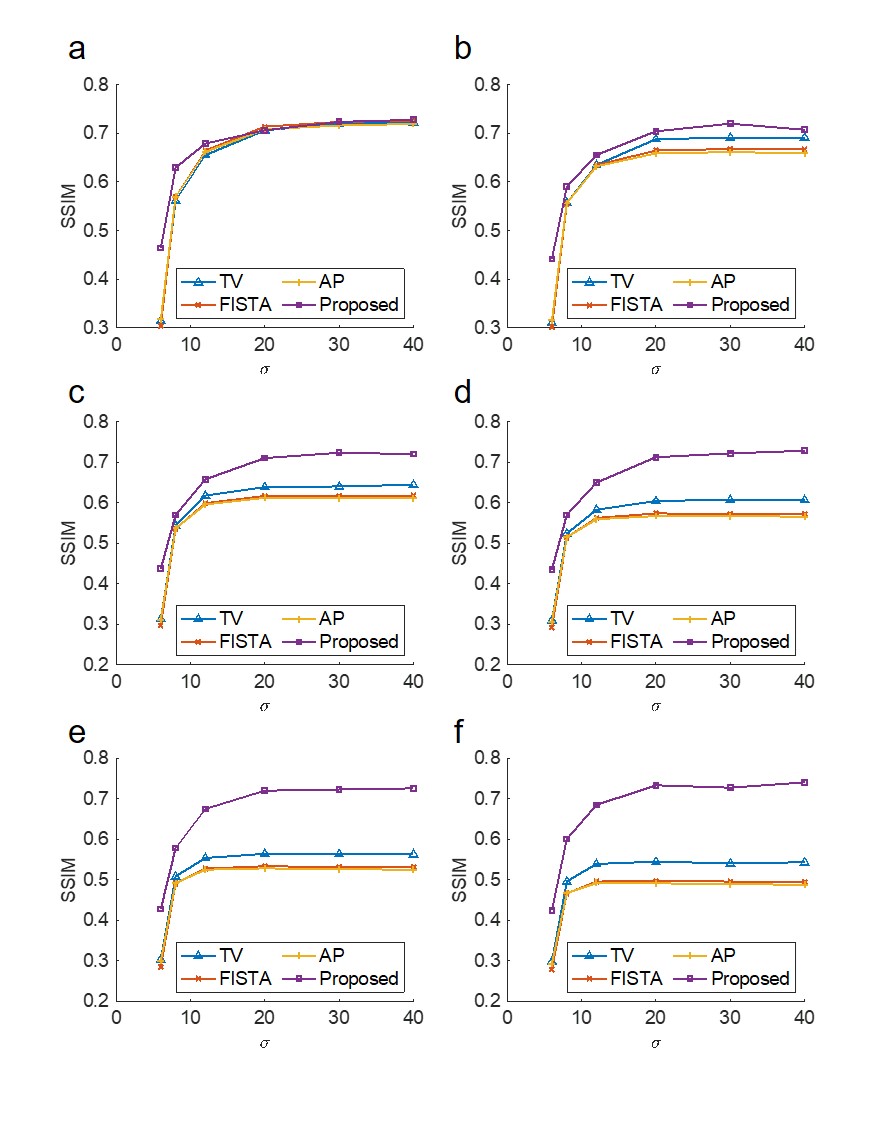


**Fig. S13** Calculated SSIM^S7^ as a function of *σ* with *r_ns_* = 0.05 **a** *r_ns_* = 0.10 **b** *r_ns_* = 0.15 **c** *r_ns_* = 0.20 **d** *r_ns_* = 0.25 **e** and *r_ns_* = 0.30 **f** for different methods including FISTA^49^, TV^50^ and AP^51^ and our proposed method.

The performance metrics of PSNR and SSIM are shown in Fig. S12 and Fig. S13, respectively. Without loss of generality, the compression ratio is set as 0.5. As shown in Fig. S12a and Fig. S13a, when the nonuniformity is small, *i.e.*, *r_ns_* = 0.05 and *σ* = 30～40, our proposed method and other conventional methods^49-51^ could achieve comparable reconstruction quality. It is reasonable since conventional methods are noise-tolerant. The slight nonuniformity can be treated as noise, which can be easily diminished. However, when the nonuniformity is relatively larger, *i.e.*, *r_ns_* = 0.10-0.30, and *σ* < 30, the reconstruction quality of conventional methods degenerates significantly. On the contrary, the performance of the proposed method could address the nonuniformity, and the obtained PSNR and SSIM are much higher than conventional reconstruction algorithms. Besides, the performance of the proposed method is robust to different degrees of nonuniformity, and the performance improvement with ACS reconstruction becomes more obvious with the increased nonuniformity.

In conclusion, these experimental comparisons prove that our method provides an elegant solution to handle different degrees of the nonuniformity existing in the CS imaging experiments, therefore lending more insight to further improve the CS imaging quality in similar THz imaging systems.

**Supplementary References:**

[S1] Yoon, J., Seol, K. H., Song, S. H. & Magnusson, R. Critical coupling in dissipative surface-plasmon resonators with multiple ports. *Optics Express* **18**, 25702-25711 (2010).

[S2] Isić, G. & Gajić, R. Geometrical scaling and modal decay rates in periodic arrays of deeply subwavelength terahertz resonators. *Journal of Applied Physics* **116**, 233103 (2014).

[S3] Isić, G., Vasić, B., Zografopoulos, D. C., Beccherelli, R. & Gajić, R. Electrically tunable critically coupled terahertz metamaterial absorber based on nematic liquid crystals. *Physical Review Applied* **3**, 064007 (2015).

[S4] Wang, H., Wu, T. X., Zhu, X. & Wu, S.-T. Correlations between liquid crystal director reorientation and optical response time of a homeotropic cell. *Journal of Applied Physics* **95**, 5502-5508 (2004).

[S5] Hestenes, M. R. Multiplier and gradient methods. *Journal of Optimization Theory and Applications* **4**, 303-320 (1969).

[S6] Boyd, S., Parikh, N., Chu, E., Peleato, B. & Eckstein, J. Distributed optimization and statistical learning via the alternating direction method of multipliers. *Foundations and Trends® in Machine Learning* **3**, 1-122 (2011).

[S7] Wang, Z., *et al.* Image quality assessment: from error visibility to structural similarity. *IEEE Transactions on Image Processing* **13**, 600-612 (2004).
